# AKTIP interacts with ESCRT I and is needed for the recruitment of ESCRT III subunits to the midbody

**DOI:** 10.1371/journal.pgen.1009757

**Published:** 2021-08-27

**Authors:** Chiara Merigliano, Romina Burla, Mattia La Torre, Simona Del Giudice, Hsiangling Teo, Chong Wai Liew, Alexandre Chojnowski, Wah Ing Goh, Yolanda Olmos, Klizia Maccaroni, Maria Giubettini, Irene Chiolo, Jeremy G. Carlton, Domenico Raimondo, Fiammetta Vernì, Colin L. Stewart, Daniela Rhodes, Graham D. Wright, Brian E. Burke, Isabella Saggio

**Affiliations:** 1 Sapienza University Dept. Biology and Biotechnology, Rome, Italy; 2 CNR Institute of Molecular Biology and Pathology, Rome, Italy; 3 Institute of Structural Biology, Nanyang Technological University, Singapore; 4 A*STAR, Developmental and Regenerative Biology, ASLR, Agency for Science, Technology and Research, Singapore; 5 A*STAR, Singapore Nuclear Dynamics and Architecture, ASLR Skin Research Labs, Agency for Science, Technology and Research, Singapore; 6 A*STAR Microscopy Platform, Research Support Centre, Agency for Science, Technology and Research, Singapore; 7 School of Cancer and Pharmaceutical Sciences, King’s College London, London, United Kingdom; 8 Organelle Dynamics Laboratory, The Francis Crick Institute, London, United Kingdom; 9 CrestOptics S.p.A., Rome, Italy; 10 University of Southern California, Molecular and Computational Biology Dept., Los Angeles, California, United States of America; 11 Sapienza University Dept. Molecular Medicine, Rome, Italy; 12 Dept. of Physiology National University of Singapore, Singapore; 13 School of Biological Sciences, Nanyang Technological University, Singapore; The University of North Carolina at Chapel Hill, UNITED STATES

## Abstract

To complete mitosis, the bridge that links the two daughter cells needs to be cleaved. This step is carried out by the endosomal sorting complex required for transport (ESCRT) machinery. AKTIP, a protein discovered to be associated with telomeres and the nuclear membrane in interphase cells, shares sequence similarities with the ESCRT I component TSG101. Here we present evidence that during mitosis AKTIP is part of the ESCRT machinery at the midbody. AKTIP interacts with the ESCRT I subunit VPS28 and forms a circular supra-structure at the midbody, in close proximity with TSG101 and VPS28 and adjacent to the members of the ESCRT III module CHMP2A, CHMP4B and IST1. Mechanistically, the recruitment of AKTIP is dependent on MKLP1 and independent of CEP55. AKTIP and TSG101 are needed together for the recruitment of the ESCRT III subunit CHMP4B and in parallel for the recruitment of IST1. Alone, the reduction of AKTIP impinges on IST1 and causes multinucleation. Our data altogether reveal that AKTIP is a component of the ESCRT I module and functions in the recruitment of ESCRT III components required for abscission.

## Author summary

To complete cell division, the bridge that links the two daughter cells needs to be cleaved. This step is carried out by a machinery named “endosomal sorting complex required for transport” (ESCRT). The dissection of this machinery is important in basic biology and for investigating diseases in which cell division is altered. AKTIP, a factor discovered to be needed for chromosome integrity, shares similarities with a component of the ESCRT machinery named TSG101. Here we present evidence that AKTIP is part of the ESCRT machinery, as TSG101. More specifically, we show that AKTIP physically interacts with members of the ESCRT machinery and forms a characteristic circular structure at the center of the bridge linking the daughter cells. We also show that the reduction of AKTIP levels causes defects in the assembly of the ESCRT machinery and in cell division. In future work, it will be interesting to investigate the association of AKTIP with cancer, because in tumorigenesis cell division is altered and since an implication in cancer has been described for TSG101 and other ESCRT factors.

## Introduction

To complete cytokinesis, cells need to cleave the intercellular bridge, a membrane structure enriched in microtubules linking the two daughter cells. This cleavage step, named abscission, is operated by the endosomal sorting complex required for transport (ESCRT) machinery [1,2]. Beyond controlling abscission, the ESCRT machinery operates in multivesicular body biogenesis, in viral budding and at the nuclear envelope to regenerate it during mitotic exit and to repair membrane discontinuities during interphase [2].

During cytokinesis, the members of the ESCRT machinery are positioned at the midbody of the intercellular bridge. The ESCRT machinery comprises the ESCRT I, II and III modules. The ESCRT I module includes TSG101, VPS37, MVB12 and VPS28 [1,3–5]. The ESCRT II module comprises VPS22, VPS25 and VPS36 [1,6]. The ESCRT I TSG101 interacts with the ESCRT associated factor SEPT9 [7] and with the ESCRT I VPS28 [8,9]. The interaction of TSG101 with SEPT9 is required for correct assembly of ESCRT II and III modules [7]. The interaction of VPS28 with the ESCRT II subunit VPS36 and the location pattern of VPS36 at the midbody suggest that it is a bridging element between ESCRT I and ESCRT III complexes [10]. CHMP2A, CHMP4B and IST1 are ESCRT III members. Together, and as an alternative to the ESCRT I/II pathway, the ESCRT associated protein ALIX contributes to ESCRT III recruitment [3,4,11].

The assembly of the ESCRT machinery at the midbody is initiated by CEP55, which recruits the ESCRT I component TSG101 and ALIX [3,5,12,13]. CEP55 positioning at the midbody depends on centralspindlin subunit MKLP1 [13]. Cytokinesis can proceed also via CEP55 independent mechanisms [14]. In *Drosophila* which has no CEP55, ALIX and TSG101 recruitment to the midbody is promoted by the centralspindlin component Pavarotti (Pav, in human MKLP1) [15].

Super-resolution microscopy has permitted the interpretation at 100–200 nanometer scale of the organization of ESCRT and ESCRT associated factors supra-molecular complexes formed at the midbody [16–19]. Such studies have shown that SEPT9, CEP55 and TSG101 form circular structures in the central area of the midbody [1,2,7,10,12,13]. The ESCRT II VPS36 organizes as a ring in early midbodies and then laterally stretches towards the constriction site [10]. The ESCRT III subunits CHMP2A, CHMP4B and IST1 are first organized as double rings at the two sides of the ESCRT I/II complex. In later stages, the organization of these ESCRT subunits evolves into spiral structures with progressively smaller diameters at the constriction site [16,19]. The reorganization of ESCRT III into spirals is thought to lead to the scission of the intercellular bridge [16,19,20].

In our previous work, we have shown that a protein named AKTIP (in humans, Peo in *Drosophila*) is organized in foci enriched at the nuclear rim in interphase cells and that a reduction in AKTIP (or Peo) results in telomeric defects, aging and cancer aggressiveness [21–25]. AKTIP shares sequence similarities with the ESCRT I member TSG101 [26,27], and has been associated with vesicle trafficking and membrane tubulation [28,29], we therefore investigated if AKTIP is an ESCRT associated factor. Here we report that in mitotic cells AKTIP locates to midbody in association with the ESCRT complex. AKTIP forms a ring in the central dark zone of the intercellular bridge, localizing closely to the ESCRT I components TSG101 and VPS28 and in proximity to ESCRT III subunits. We further show that AKTIP recruitment to the midbody is dependent on MKLP1 and independent of CEP55, TSG101 and ALIX. Biochemical data show that AKTIP interacts with the ESCRT I subunits VPS28 and TSG101. AKTIP and TSG101 are needed for the recruitment of the ESCRT III subunits CHMP4B and IST1 to the midbody and AKTIP loss generates cytokinesis defects. Altogether these data reveal an association of AKTIP with the ESCRT machinery, namely the ESCRT I subcomplex, and a role played by AKTIP during abscission based on its direct interaction with VPS28 and its action on ESCRT III components.

## Results

### AKTIP forms a ring in the dark zone of the intercellular bridge that links the two daughter cells in cytokinesis

**Fig 1:**
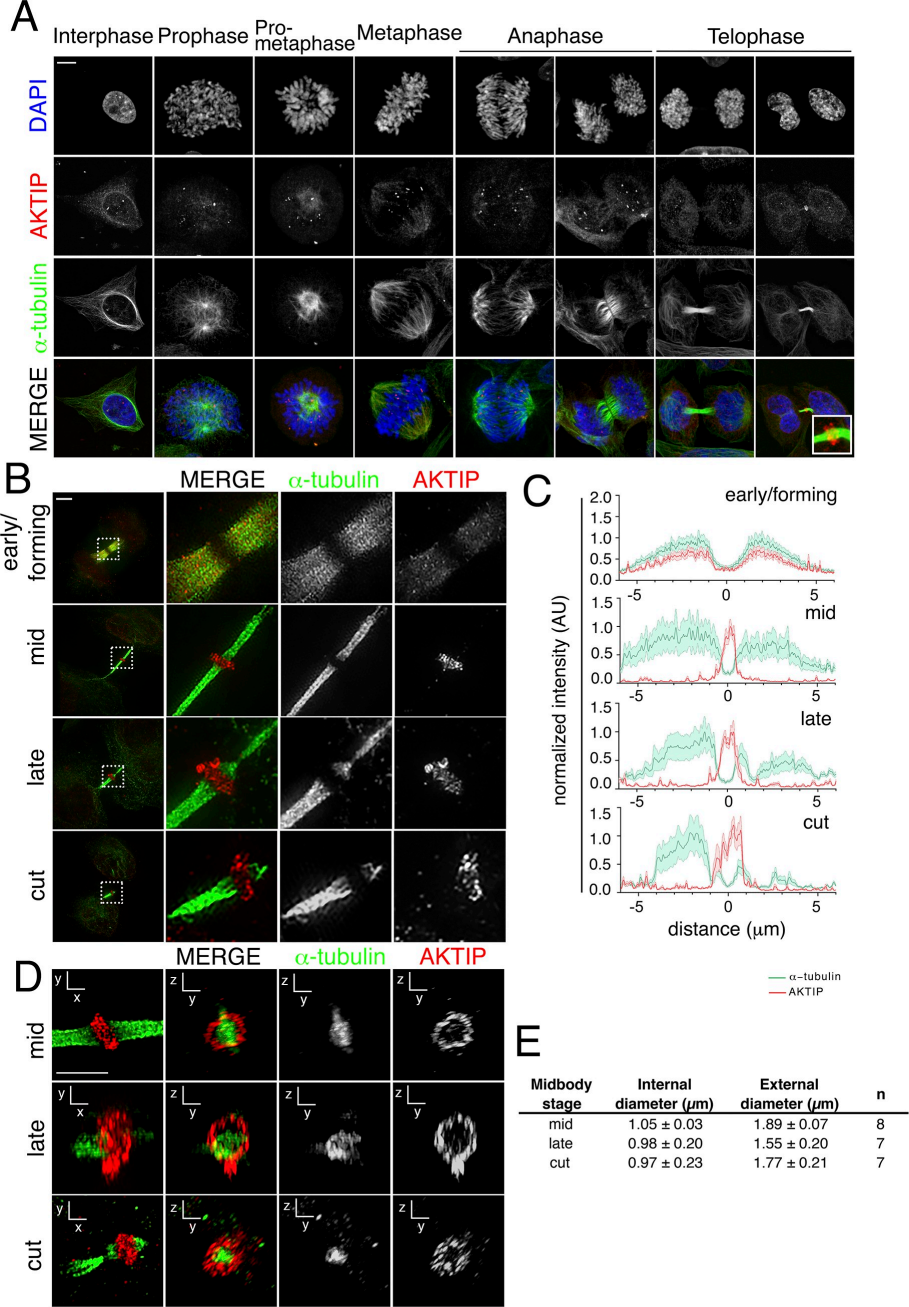
AKTIP localizes as a ring at the dark zone of the midbody. **(A)** Confocal immunofluorescence images showing the distribution of AKTIP during mitosis and cytokinesis. HeLa cells were stained with anti-AKTIP (red), anti-α-tubulin (green) and DAPI to visualize DNA (blue). Scale bar, 5μm. **(B)** Representative 3D-SIM images of cells observed for AKTIP at the midbody in early, mid, late and cut stages. HeLa cells were stained with α-tubulin (green) and AKTIP (red) antibodies. Scale bar, 5μm. **(C)** Representative fluorescence intensity profile plotted for AKTIP and α-tubulin along the midbody at different stages; early/forming (n = 6), mid (n = 7), late (n = 8), cut (n = 8). (**D-E)** Representative 3D-SIM images of AKTIP ring (D) and relative measurements (E). Size of AKTIP structure measured in mid (n = 8), late (n = 7) and cut (n = 7) midbodies. Scale bar, 2.5μm.

To gain insights into the role of AKTIP, we analyzed its spatiotemporal distribution from interphase to late telophase. HeLa cells were immunostained for DNA (DAPI), α-tubulin and endogenous AKTIP and imaged by confocal microscopy. In interphase, AKTIP is detectable as discrete and abundant foci at the nuclear rim and within the nucleus. During the initial phases of mitosis, AKTIP moves along the microtubules and on the spindle midzone during anaphase. In late telophase, AKTIP accumulates on the intercellular bridge at the center of the midbody. At this stage, the AKTIP punctate signal is also visible at the reforming nuclear rim and in the nucleoplasm (Fig 1A).

To validate the specificity of the localization of AKTIP to the midbody, we compared the immunostaining obtained using the monoclonal anti-AKTIP antibody (2A11 WH006400M2 Sigma-Aldrich, Figs 1 and S1A) to that obtained with a polyclonal antibody (HPA041794 Sigma-Aldrich; S1A Fig). We also analyzed the localization of exogenously expressed AKTIP using an anti-FLAG antibody in cells transiently transfected with an AKTIP-FLAG expressing construct (S1B Fig). In all cases, we observe a clear AKTIP signal at the center of the intercellular bridge. Next, we produced HeLa cells with a stable knock down of AKTIP using lentiviral mediated RNA interference (shAKTIP). As compared to HeLa cells stably modified with a lentiviral vector containing a scramble hairpin interfering sequence (shctr), we observe in shAKTIP cells the loss of the endogenous AKTIP signal at the midbody (S1C Fig), the depletion of protein expression (S1D Fig) and reduction of mRNA expression (S1E Fig).

To obtain high resolution information of the organization of AKTIP at the midbody, we used 3D structured illumination microscopy (3D-SIM), which delivers ~120nm resolution [30]. Cells were stained with anti-AKTIP and anti-α-tubulin antibodies and 3D-SIM images were reconstructed (Fig 1B and S1 and S2 Movies). We subdivided midbody stages into early, mid, late and cut, as previously described [7,16]. In the early/forming stage, the midbodies have the largest diameter and the tube is symmetric with respect to the central dark zone (or Flemming body). In mid-stage midbodies the microtubules form a structure that is still symmetric with respect to the dark zone but has a smaller diameter. Late stage midbodies are recognizable by their asymmetry and for the presence of the constriction site. In the early/forming stage, AKTIP is detected as a set of multiple spots on the microtubules of the midbody (Fig 1B). Image quantification shows similar distribution for α-tubulin and AKTIP signals (Fig 1C). During the mid- and late-stage, AKTIP is organized as a ring structure around the central section of the microtubule tube, while staining on the midbody arms is almost entirely absent (Fig 1B and 1C). In the mid-stage, the AKTIP signal reaches its maximal intensity in the dark zone of the midbody where α-tubulin detection is at its lowest. In mid-stage, the average internal diameter of the AKTIP ring is equal to measures 1.05±0.03μm, and the external diameter is 1.89±0.077μm (Fig 1D and 1E). Comparison of our data to available measurements based on super-resolution microscopy of ESCRT factors indicates that the AKTIP ring size is similar to that formed by ESCRT I subunit TSG101 and by ESCRT II subunit VPS36, and slightly larger than that calculated for the ESCRT III module components [10,16,19].

These data taken together demonstrate a physical organization of AKTIP at the midbody similar to that of components of the ESCRT machinery.

### AKTIP interacts with the ESCRT I subunit VPS28

**Fig 2:**
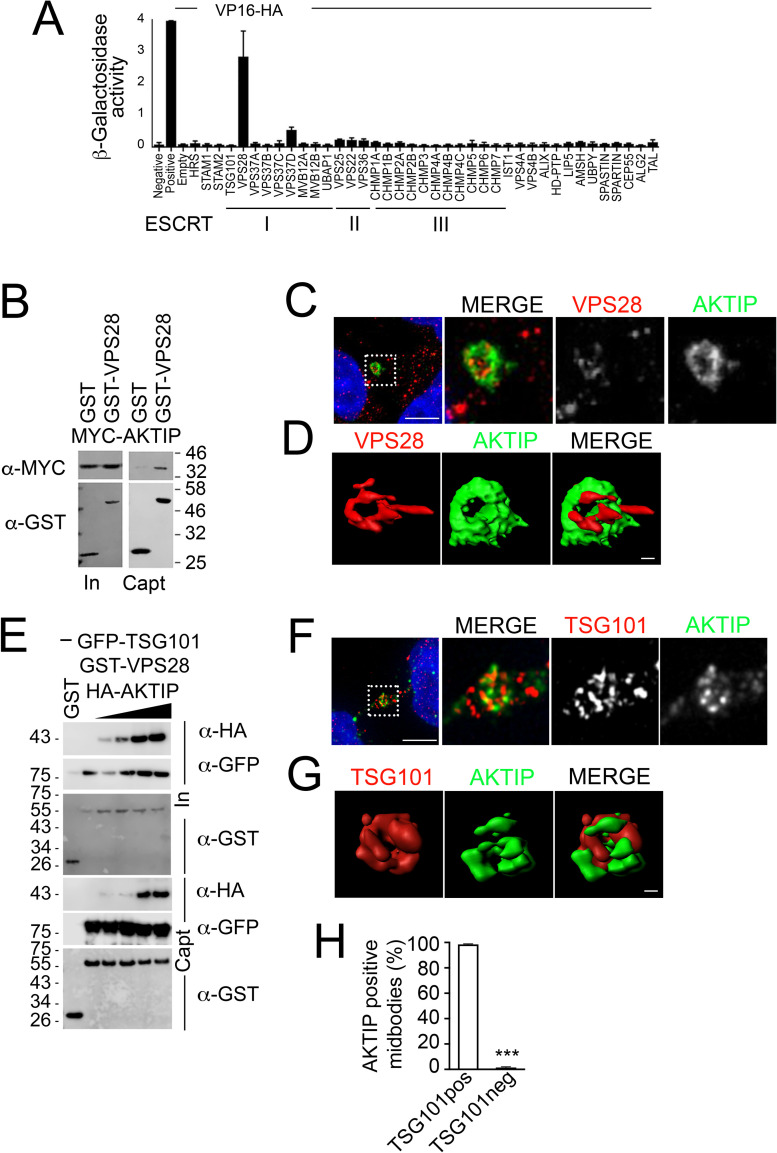
AKTIP is associated with the ESCRT I subunits VPS28 and TSG101. **(A)** AKTIP fused to the Gal4 DNA binding domain was tested for interactions with the human components of ESCRT I, II, III, and ESCRT associated proteins fused to the VP16 activation domain by yeast two-hybrid assay. Error bars indicate the SEM from the mean of triplicate measurements. **(B)** Western blotting showing that AKTIP interacts with GST-VPS28 but not with GST alone. Cells were transfected with plasmids encoding the indicated fusion proteins. Purified VPS28-GST or GST alone were used to pull down interacting proteins; cell lysates and glutathione-bound fractions were then analyzed with MYC antisera. GST-pull down was repeated three times. **(C)** Spinning disk microscopy images of AKTIP (green) and VPS28 (red). Scale bar, 5μm. **(D)** 3D rendering of spinning disk imaging as in (C) showing that AKTIP and VPS28 are in proximity at midbody. VPS28 in late midbodies displays also an asymmetric protruding element. Scale bar, 0.5 μm. **(E)** Western blotting showing that HA-AKTIP, GFP-TSG101 and GST-VPS28 are captured together in GST pull down experiment. Cells were co-transfected with a fixed amount of plasmid encoding GFP-TSG101 (500 ng) and GST-VPS28 (1000 ng) and increasing amounts of HA-AKTIP (0, 50, 100, 500 or 1000 ng) encoding plasmid. Cell lysates and GST pull down fractions were then analyzed with GST, GFP, HA antisera. GST-pull down was repeated three times. **(F)** Spinning disk microscopy images of AKTIP (green) and TSG101 (red). Scale bar, 5μm. **(G)** 3D rendering of spinning disk imaging as in (E) showing that AKTIP and TSG101 are near each other. Scale bar, 0.5 μm. **(H)** Quantification of the percentage of midbodies positive both for AKTIP and TSG101 showing that the two proteins are concomitantly present at the midbody. For results in (H) at least 100 midbodies per condition were counted.

To investigate the biochemical association of AKTIP with the ESCRT machinery we carried out a yeast two hybrid screen on ESCRT I, II, III subunits and on the ESCRT associated factors. Yeast cells were co-transformed with a plasmid encoding AKTIP fused to the Gal4 DNA-binding domain, in combination with plasmids encoding ESCRT or ESCRT associated factors fused to the VP16 activation domain and the LacZ activity in co-transformants was measured. We observe that AKTIP significantly interacts with the ESCRT I subunit VPS28 (Fig 2A).

To validate the interaction between AKTIP and VPS28 in mammalian cells, VPS28 was cloned as a GST-fusion and AKTIP as a MYC- or HA-tagged fusion. 293T cells were co-transfected with control GST or GST-VPS28 and either MYC-AKTIP or HA-AKTIP. Pull down assays followed by Western blotting show that AKTIP co-precipitates with VPS28 (Figs 2B and S2). The co-precipitation signal is observed with both MYC- and HA-tagged AKTIP. GST alone, as expected, does not interact with AKTIP (Figs 2B and S2).

We next analyzed in vivo the co-localization of AKTIP and VPS28. Spinning disk analyses and image reconstruction of HeLa cells stained with anti-AKTIP and anti-VPS28 antibodies show that the two signals are in proximity at the midbody (Fig 2C and 2D). The signal of VPS28 in addition to its localization at the center, close to AKTIP, displays an asymmetric tubular protruding element (Fig 2C and 2D).

Since TSG101 is known to bind VPS28 [6,31–34], we next asked whether VPS28, AKTIP and TSG101 could be present in common complexes. To address this question, we co-expressed in 293T cells GST or GST-VPS28 with GFP-TSG101 and increasing concentrations of HA-AKTIP and performed a pulldown assay. The results of this experiment show that HA-AKTIP, GFP-TSG101 and GST-VPS28 are captured together, suggesting the possibility of either a complex including AKTIP, VPS28 and TSG101 or of AKTIP/VPS8 and TSG101/VPS28 independent complexes (Fig 2E). To further explore this point, we investigated whether endogenous AKTIP and TSG101 are concomitantly detected at the midbody in vivo. To this end, we immunostained HeLa cells with anti-AKTIP and anti-TSG101 antibodies. 3D reconstruction of the images shows that AKTIP and TSG101 closely localize (Fig 2F and 2G and S3 Movie). Furthermore, co-immunofluorescence quantification of AKTIP and TSG101 shows that 99% (n = 100) of the midbodies that are positive for AKTIP are also positive for TSG101 (Fig 2H).

Taken together, these data show that VPS28 can be in complex with both TSG101 and AKTIP. In vivo, AKTIP and TSG101 and AKTIP and VPS28 are concomitantly present in proximity at the center of the midbody.

### The UEV domain of AKTIP interacts with the N-terminus of VPS28

**Fig 3:**
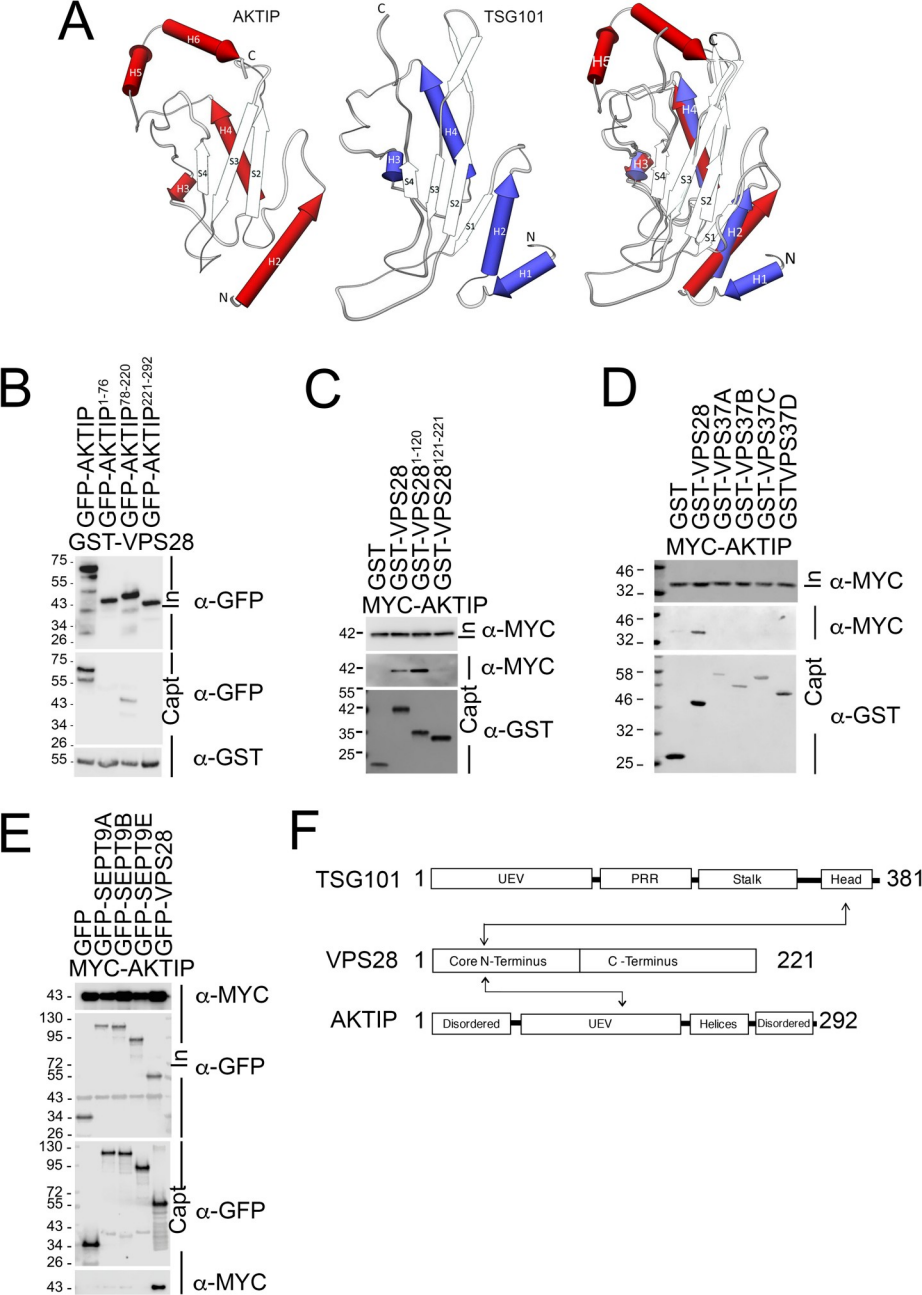
The central region of AKTIP interacts with the N-terminus of VPS28. **(A)** Superimposing of AKTIP model on TSG101 X-ray solved structure highlights similarities in the central region and two main different elements outside of it. Namely, the AKTIP central UEV domain presents two C-terminal helices (H5 and H6), absent in TSG101; TSG101 contains two N-terminal helices (H1 and H2), while AKTIP only one (H2). **(B-C)** Western blotting showing that central UEV region of AKTIP interacts with GST-VPS28 (B) and that AKTIP interacts with N-terminal domain of VPS28 (C). Cells were transfected with plasmids encoding the indicated fusion proteins and the AKTIP protein N-terminal (1–76 aa, NT), central domain (78–220 aa), C-terminal (221–292 aa) fragments. Purified VPS28-GST, or N-terminal (1–120 aa, NT) VPS28, or C-terminal (121-221aa, CT) or GST alone were used to pull down interacting proteins; cell lysates and glutathione-bound fractions were then analyzed with GST, GFP or MYC antisera. GST-pull downs were repeated three times. **(D)** Western blotting showing that AKTIP does not interact with VPS37 A to D isotypes and confirming its interaction with VPS28. Cells were transfected with plasmids encoding the indicated fusion proteins. Purified VPS28-GST or VPS37(A-D)-GST or GST alone were used to pull down interacting proteins; cell lysates and glutathione-bound fractions were then analyzed with MYC antisera. GST-pull downs were repeated two times. **(E)** Western blotting showing that AKTIP does not interact with SEPT9 (A, B and E) and confirming its interaction with VPS28. Cells were transfected with the indicated fusion proteins. Purified SEPT9 (isoforms A, B and E)-GFP, VPS28-GFP or GFP alone were used to trap interacting proteins; then cell lysates and GFP-trapped fractions were analyzed with MYC antisera. GFP-TRAP were repeated three times. **(F)** Schematic representation of the interacting regions of AKTIP and TSG101 with VPS28. UEV (Ubiquitin E2 variant domain); TSG101 PRR (Proline Rich Region).

AKTIP and TSG101 sequences contain a ubiquitin E2 variant (UEV) domain, which in AKTIP is in its central region (aa 78–220) (S3A Fig). To compare AKTIP and TSG101, we superimposed the AKTIP three-dimensional model [21] to the X-ray structure of TSG101 [35], finding a root mean square deviation over equivalent C_α_ positions equal to 1.9 Å (Fig 3A). Between TSG101 and AKTIP there are, however, important differences. Most significantly, the AKTIP UEV domain presents two C-terminal helices (reported in Fig 3A as H5 and H6) that are absent in TSG101. We also observe, that while the residues allowing TSG101 UEV domain to bind PTAP-motifs project off into solution [26], analogous sites in AKTIP are predicted to be buried by these C-terminal helices. AKTIP differs from TSG101 in an additional way: TSG101 contains two N-terminal helices (H1 and H2), where AKTIP contains only one (H2) (Fig 3A).

To define the AKTIP region implicated in the interaction with VPS28, we produced three GFP-tagged AKTIP truncations, including the N-terminus (aa 1–76), the C-terminus (aa 221–292) or the UEV (aa 78–220) portions of AKTIP. 293T cells were co-transfected with the different AKTIP constructs together with full length GST-VPS28. Pulldown experiments demonstrate that the central region of AKTIP interacts with VPS28 (Fig 3B). To further define the interaction, we produced truncations of VPS28 corresponding to the N- and C-terminus of the protein (aa 1–120 and 121–221, respectively). The pulldown experiments show that the N-terminus of VPS28 is primarily responsible for the interaction with AKTIP (Fig 3C).

To extend the characterization of AKTIP in relation with the ESCRT subunits, we tested AKTIP on the ESCRT I subunits VPS37A to D and on the ESCRT II subunits VPS25, VPS36 and VPS22. To this end GST- or GFP-tagged ESCRT subunits were tested on MYC-tagged AKTIP. Pull down assay shows no significant interaction between AKTIP and ESCRT I VPS37A to D or ESCRT II VPS25, VPS36 and VPS22 (Figs 3D and S3B). On the other hand, the interaction between VPS28 and AKTIP was confirmed. These results are consistent with the yeast two hybrid screen data in which the values corresponding to the interaction between AKTIP and ESCRT factors are below or at the limit of significancy bar that between AKTIP and VPS28 (Fig 2A).

Given the similarity between AKTIP and TSG101, we explored the possibility of an interaction between AKTIP and SEPT9. This idea comes from the fact that an interaction between SEPT9 and TSG101 was previously described and assigned to two N-terminal PTAP motifs in SEPT9 [7]. We used GFP-tagged SEPT9 A, B and E isoforms and tested them on MYC-tagged AKTIP. We did not observe an interaction between SEPT9 and AKTIP, while the interaction between GFP-VPS28 and MYC-AKTIP was furtherly confirmed (Fig 3E).

Together these data show that AKTIP interacts with the ESCRT I module and shares with the ESCRT I subunit TSG101 the ability to bind the N-terminus of VPS28. However, at the condition tested and differently from TSG101, AKTIP binds VPS28 through its UEV domain and does not bind SEPT9 (Fig 3F).

### The AKTIP ring localizes in between the ESCRT III circular structures

The ESCRT I subunits require for the execution of abscission the ESCRT III module. To evaluate the relationship between AKTIP and the ESCRT III module, we carried out 3D-SIM co-analyzation studies of AKTIP and of the ESCRT III components CHMP4B, CHMP2A and IST1, for which the localization has been previously defined using super-resolution microscopy [16,19,36].

**Fig 4:**
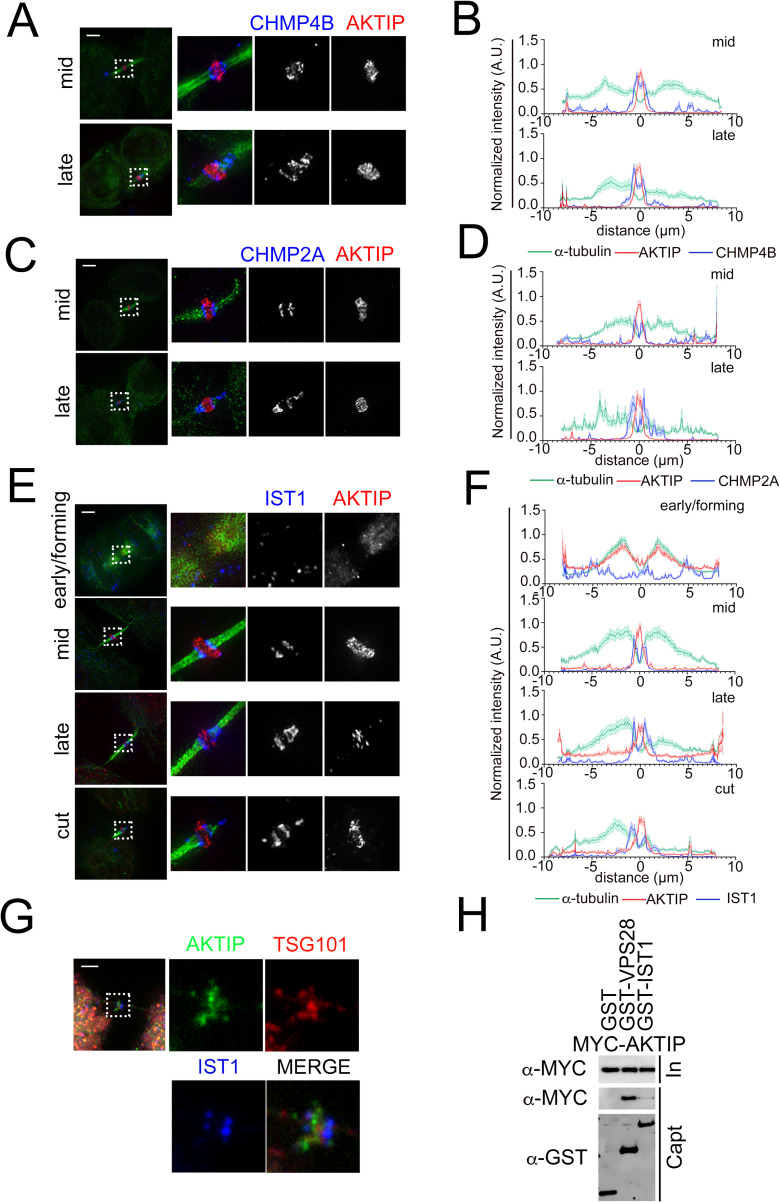
The supra-molecular structure of AKTIP is flanked by the rings formed by the ESCRT III components. **(A, C, E)** 3D-SIM images of ESCRT III components CHMP4B (A), CHMP2A (C), IST1 (E) and AKTIP in HeLa cells. Staining with antibodies against ESCRT III (blue), AKTIP (red) and α-tubulin (green). **(B, D, F)** Representative fluorescence intensity profile plotted for AKTIP, ESCRT III subunits and α-tubulin along the midbodies at different stages. (B) mid, n = 6; late, n = 4. (D) mid, n = 6; late, n = 3. (F) early/forming, n = 6; mid, n = 7; late, n = 8; cut, n = 6. **(G)** Spinning disk microscopy images of IST1 (blue), AKTIP (green) and TSG101 (red). Scale bars, 2.5μm. **(H)** Western blotting showing that AKTIP interacts with GST-VPS28, but not with GST-IST1 or GST alone. Cells were transfected with plasmids encoding the indicated fusion proteins. Purified GST-VPS28 or GST-IST1 or GST alone were used to pull down interacting proteins; cell lysates and glutathione-bound fractions were then analyzed with MYC and GST antisera. GST-pull downs were repeated three times.

Co-staining of AKTIP and CHMP4B (Fig 4A and 4B) or CHMP2A (Fig 4C and 4D) in HeLa cells shows that the ring shaped supra-molecular structure formed by AKTIP is located in between the two rings composed by these ESCRT III subunits.

We next analyzed the proximity of AKTIP to IST1. This analysis shows that in early forming midbodies neither AKTIP nor IST1 are yet organized in a supra-molecular structure. In the mid-stage, AKTIP and IST1 are detected as ring shaped structures. Two IST1 rings flank the central, single, larger, and thicker AKTIP ring at the midbody (Fig 4E and 4F and S4 Movie). In late-to-cut stages, IST1 spirals become apparent on the asymmetric tubulin bridge, while AKTIP is detected at the center of the midbody (Fig 4E and 4F and S5 Movie).

Triple staining of AKTIP, TSG101 and IST1 in HeLa cells shows that while AKTIP and TSG101 localize in close proximity, the IST1 rings flank the AKTIP-TSG101 signal (Fig 4G). In agreement with this observation, co-immunoprecipitation experiments show that GST-IST1 does not pull down MYC-AKTIP, while, at the same experimental conditions GST-VPS28 pulls down MYC-AKTIP (Fig 4H). These results are in agreement with the data obtained with the yeast two hybrid screening, that show no interaction between AKTIP and any of the ESCRT III proteins (Fig 2A).

These results taken together show that the AKTIP circular supra-molecular structure is associated with the ESCRT I module that includes TSG101 and VPS28, and localizes in proximity, albeit not coincidently, with ESCRT III subunits IST1, CHMP2A and CHMP4B when these organize into double rings in the central area of the midbody.

### AKTIP recruitment to the midbody does not require CEP55, TSG101, VPS28 and ALIX

**Fig 5:**
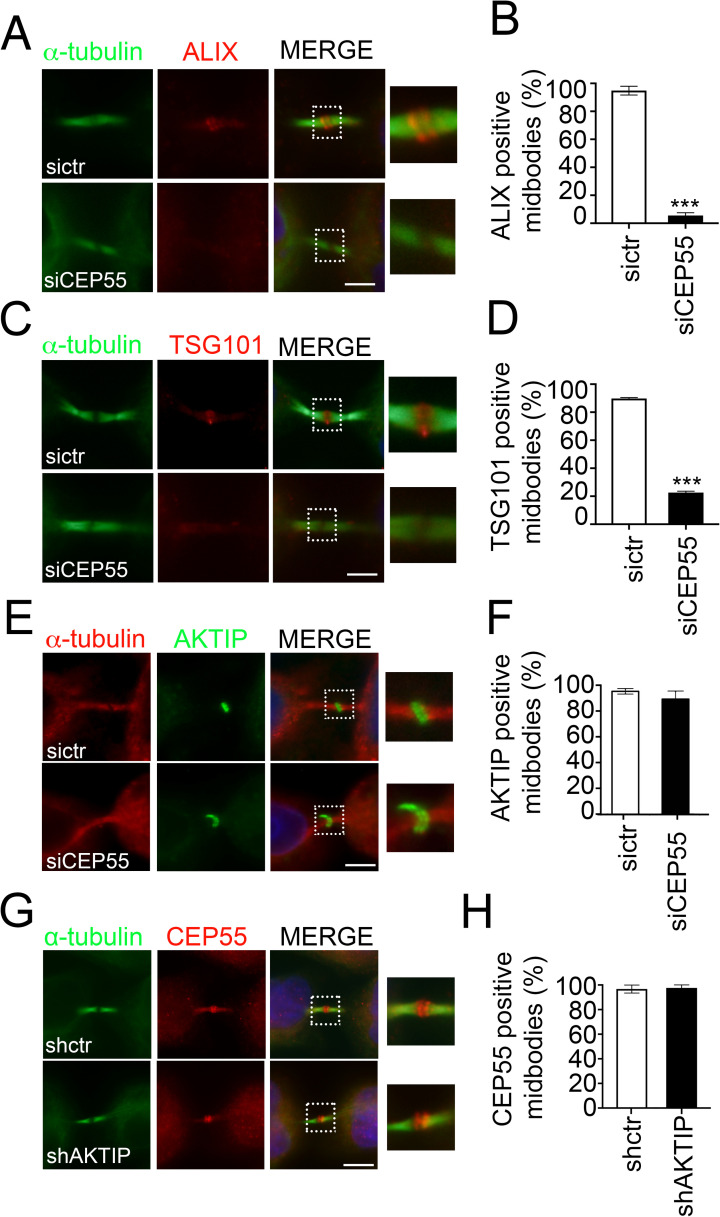
AKTIP recruitment to the midbody is CEP55 independent. **(A-B)** Representative images and quantification of sictr and siCEP55 HeLa cells stained for ALIX (red) and α-tubulin (green). **(C-D)** Representative images and quantification of sictr and siCEP55 HeLa cells stained for TSG101 (red) and α-tubulin (green). **(E-F)** Representative images and relative quantification of sictr and siCEP55 HeLa cells stained for AKTIP (green) and α-tubulin (red). **(G-H)** Representative images and relative quantification of shctr and shAKTIP HeLa cells stained for CEP55 (red) and α-tubulin (green). For results in (B, D, F and H) at least 100 midbodies per condition were counted. Scale bar, 5μm.

Since in mammalian cells at the earliest stages of abscission, CEP55 controls the recruitment of TSG101 and of the TSG101 associated factor ALIX [3,5,12,13], we next investigated whether AKTIP recruitment to the midbody depended on CEP55. To address this question, we produced CEP55 depleted cells by transient RNA interference (siCEP55) (S4A and S4B Fig). Consistently with published data [13,37], we observe that siCEP55 cells are characterized by multinucleation (S4C and S4D Fig). As expected, the recruitment of TSG101 and ALIX to the midbody is significantly reduced in siCEP55 cells (Fig 5A–5D). By contrast, the number of AKTIP positive midbodies is not reduced (Fig 5E and 5F). We next performed the reverse experiment staining for CEP55 in HeLa cells with stable knock down of AKTIP (shAKTIP, S1C–S1E Fig). The quantification of immunofluorescence images shows that in shAKTIP cells, the number of CEP55 positive midbodies is the same as in the control cell line (shctr, Fig 5G and 5H). Altogether these data show that AKTIP recruitment to the midbody is independent of CEP55. Consistently with this observation, we observe no interaction of AKTIP with CEP55 in the yeast two hybrid screening (Fig 2A), and the residues 158–162 of TSG101 (S3A Fig), needed for CEP55 interaction [3,5,12], are not present in AKTIP. These data also indirectly indicate that the recruitment of AKTIP is independent of the presence of ALIX and TSG101.

**Fig 6:**
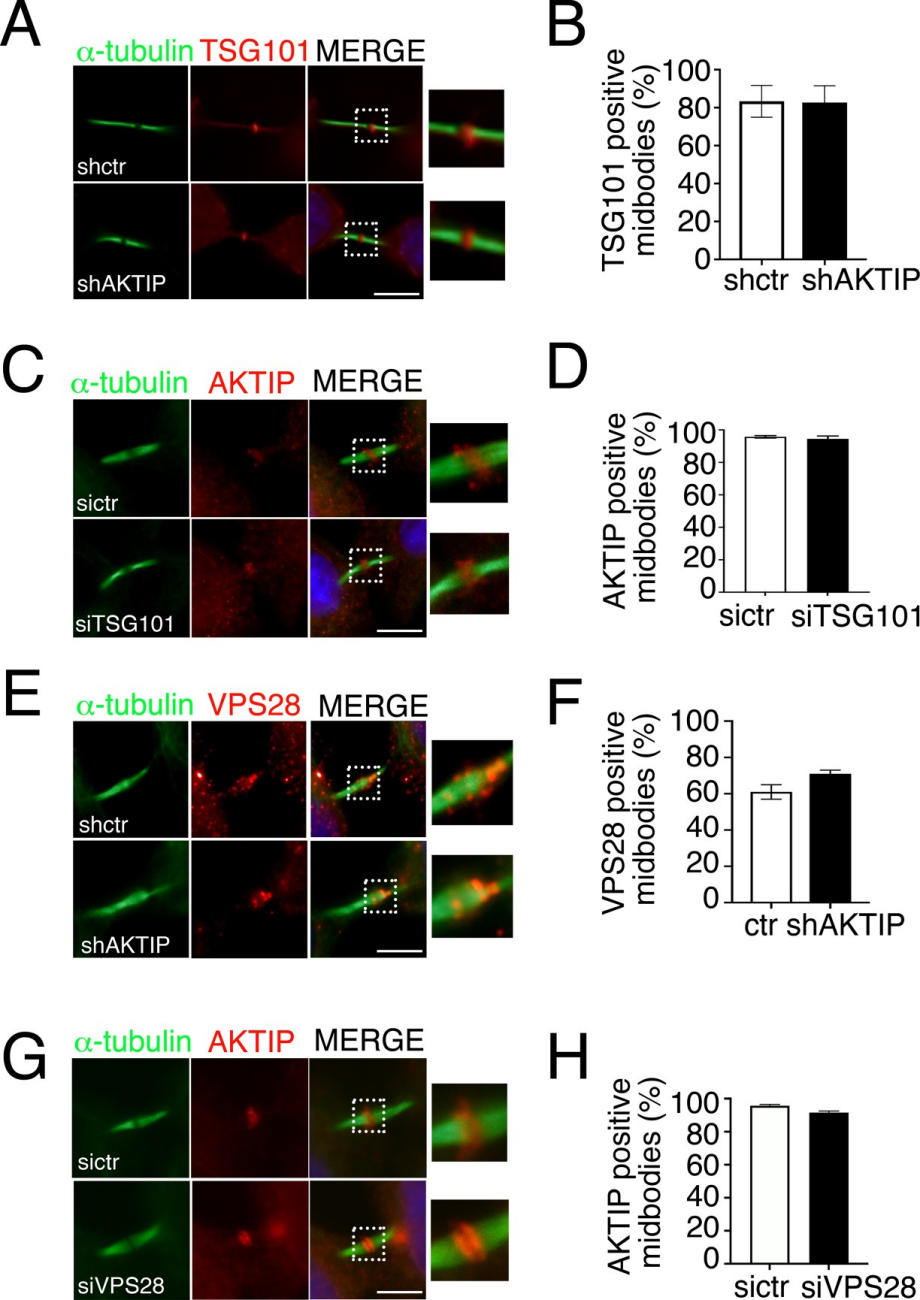
AKTIP, TSG101 and VPS28 are independently recruited to the midbody. **(A-B)** Representative images and relative quantification of shctr and shAKTIP HeLa cells stained for TSG101 (red) and α-tubulin (green) showing that TSG101 is present at the midbody in shAKTIP cells. **(C-D)** Representative images and relative quantification of sictr and siTSG101 HeLa cells stained for AKTIP (red) and α-tubulin (green) showing that AKTIP is present at the midbody in siTSG101 cells **(E-F)** Representative images and relative quantification of shctr and shAKTIP HeLa cells stained for VPS28 (red) and α-tubulin (green) showing that VPS28 is present at the midbody in shAKTIP cells. **(G-H)** Representative images and relative quantification of sictr and siVPS28 HeLa cells stained for AKTIP (red) and α-tubulin (green) showing that AKTIP is present at the midbody in siVPS28 cells. For results in (B, D, F and H) at least 100 midbodies per conditions were counted. Scale bars, 5μm.

To validate this last point, i.e. the independence of AKTIP from TSG101 and ALIX extrapolated from the data obtained in siCEP55 cells, we analyzed by immunofluorescence TSG101 in shAKTIP cells. This analysis shows that the level of TSG101 positive midbodies is not dependent on AKTIP (Fig 6A and 6B). We next monitored the reverse condition, that is the recruitment of AKTIP to the midbody siTSG101 cells, and we did not observe a significant loss in the recruitment of AKTIP (Figs 6C and 6D and S5A and S5B).

We next monitored by immunofluorescence the midbodies for the presence of ALIX in shAKTIP cells and reverse. We observe that AKTIP loss does not impact on ALIX recruitment to the midbody and viceversa (S5C–S5H Fig).

To complete the picture of the elements playing a role in the assembly of the early members of the ESCRT complex and given the biochemical interaction of AKTIP with VPS28, we checked whether VPS28 was affected in shAKTIP cells. Immunofluorescence analysis shows no significant differences of VPS28 in shAKTIP cells as compared to controls (Fig 6E and 6F). The reverse experiment, in which VSP28 expression was reduced, showed that AKTIP is recruited to the midbody in siVPS28 cells as in controls (Figs 6G and 6H and S5I and S5J).

Altogether, these results show that the recruitment of AKTIP to the midbody can happen independently of CEP55, TSG101, ALIX and VPS28.

### The recruitment of AKTIP to the midbody requires MKLP1

We next investigated whether AKTIP recruitment to the midbody was dependent on MKLP1. The idea for this came from data showing that in *Drosophila* the recruitment to the midbody of TSG101 and ALIX can be independent of CEP55, while it is promoted by the centralspindlin component Pav (MKLP1 in mammals) [15]. We also considered the evidence that in mouse primary fibroblasts cytokinesis happens in a CEP55 independent, but MKLP1 dependent manner [14].

**Fig 7:**
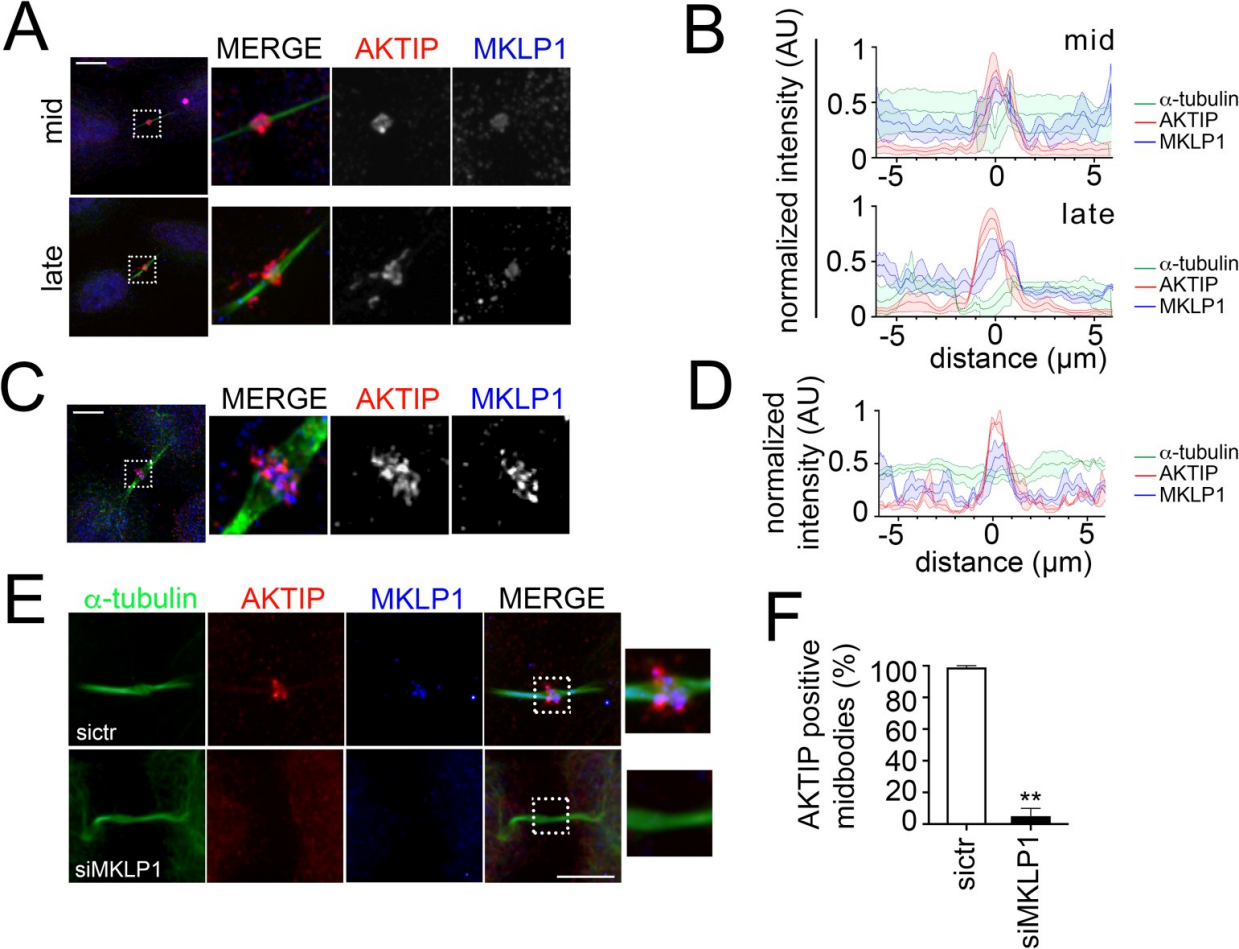
AKTIP recruitment to the midbody is MKLP1 dependent. **(A)** Spinning disk microscopy images of MKLP1 (blue), AKTIP (red) and α-tubulin (green). **(B)** Representative fluorescence intensity profile plotted for AKTIP, MKLP1 and α-tubulin along the midbodies at different stages; mid, n = 3; late, n = 3. **(C)** MSIM images of MKLP1 (blue), AKTIP (red) and α-tubulin (green). **(D)** Representative fluorescence intensity profile plotted for AKTIP, MKLP1 and α-tubulin along mid midbodies from (C); n = 3. **(E)** Representative images of sictr and siMKLP1 HeLa cells stained for AKTIP (red), MKLP1 (blue) and α-tubulin (green) showing that AKTIP recruitment is MKLP1 dependent. **(F)** Quantification of AKTIP positive midbodies in sictr and siMKLP1 cells. For results in (F) at least 25 midbodies were counted for each sample. Scale bars, 5μm.

To analyze the association of AKTIP and MKLP1, firstly we performed co-immunofluorescence and analyzed by confocal imaging the relative spatiotemporal distribution of the two factors. We observe that AKTIP and MKLP1 have similar distributions (Fig 7A and 7B). To obtain higher resolution images and assess proximity, we analyzed by Multispot-SIM (MSIM) mid-stage midbodies. The analysis of the images and their quantification shows that MKLP1 and AKTIP signals are braided (Fig 7C and 7D).

As a second step, to evaluate if the recruitment of AKTIP was MKLP1 dependent, we analyzed AKTIP localization at the midbody in siMKLP1 cells (S6A and S6B Fig). The quantification of the images shows a specific drop of AKTIP positive midbodies in siMKLP1 cells as compared to control cells (Fig 7E and 7F).

In summary, these data provide evidence that MKLP1 not only localizes in close proximity to AKTIP but also, importantly, is needed for the recruitment of AKTIP to the midbody.

### AKTIP contributes to the recruitment of CHMP4B and of IST1

The ESCRT I module contributes to the assembly of ESCRT III modules [4,32]. Therefore, our next step was to dissect the impact of AKTIP on the ESCRT III module. We focused on CHMP4B, a pivotal constituent of the ESCRT III, and on IST1, which is an effector of the abscission process [38].

**Fig 8:**
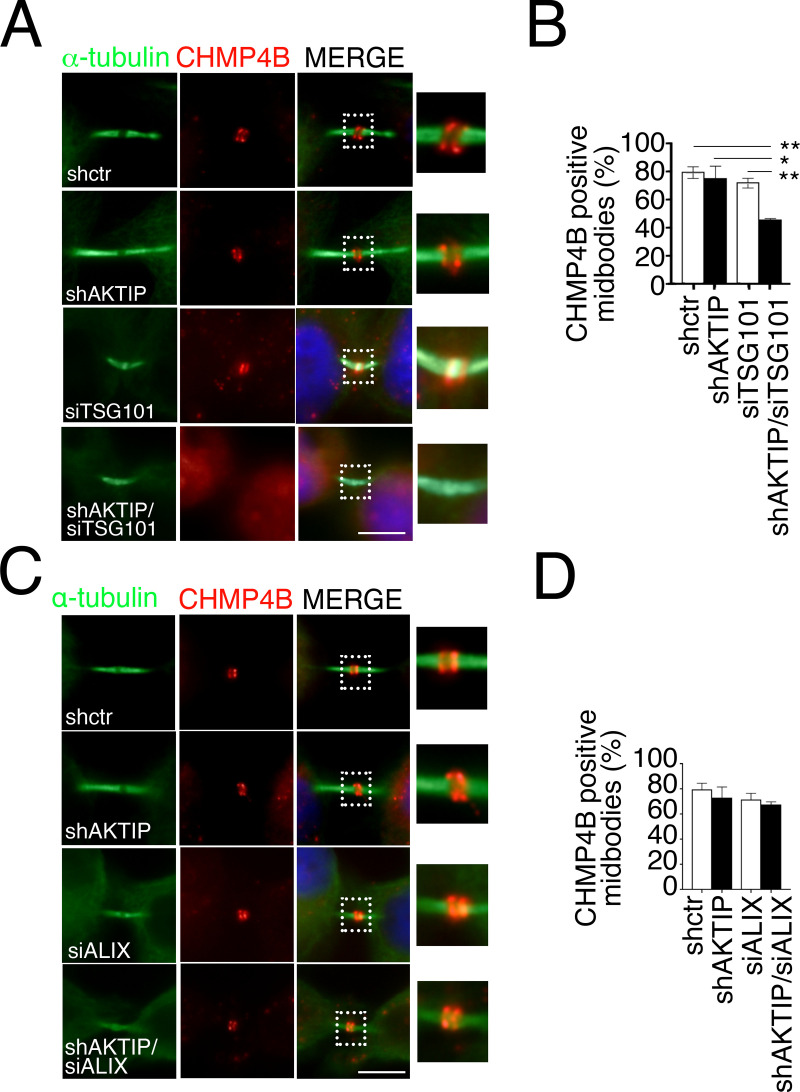
AKTIP cooperates with TSG101 but not with ALIX for CHMP4B recruitment. **(A-B)** Representative images of shctr, shAKTIP, siTSG101 and shAKTIP/siTSG101 HeLa cells stained for CHMP4B (red) and α-tubulin (green) and relative quantification. **(C-D)** Representative images of shctr, shAKTIP, siALIX and shAKTIP/siALIX HeLa cells stained for CHMP4B (red) and α-tubulin (green) and relative quantification. For results in (B and D) at least 100 midbodies per condition were counted. Scale bars, 5μm.

We firstly analyzed the ESCRT III subunit CHMP4B in shAKTIP cells by immunofluorescence. The data show that the number of CHMP4B positive midbodies in shAKTIP cells are comparable to control cells, revealing that AKTIP depletion alone is not sufficient to affect CHMP4B recruitment to the midbody (Fig 8A and 8B). Along with this, by evaluating CHMP4B at the midbody, we observed no evident abnormality, and the organization of CHMP4B into cones (spirals) and rings was comparable in shAKTIP cells and in controls (S7A Fig).

We next asked whether AKTIP would influence CHMP4B by cooperating with TSG101. We produced siTSG101 and shAKTIP/siTSG101 cells by transient transfection of siTSG101 in control and shAKTIP cells, respectively. siTSG101 causes a reduction of TSG101 positive midbodies in both conditions (S7B and S7C Fig). In line with previous data [32], siTSG101 cells display control levels of the ESCRT III member CHMP4B (Fig 8A and 8B). By contrast, shAKTIP/siTSG101 cells display significantly reduced levels of CHMP4B positive midbodies, indicating that they cooperate for recruitment of this ESCRT III subunit to the midbody (Fig 8A and 8B).

We next analyzed by immunofluorescence the presence of CHMP4B in either shAKTIP cells or in co-depleted shAKTIP, siALIX cells. We observe that the siALIX alone does not significantly affect CHMP4B recruitment, neither it does in combination with AKTIP reduction (Figs 8C and 8D and S7D and S7E).

**Fig 9:**
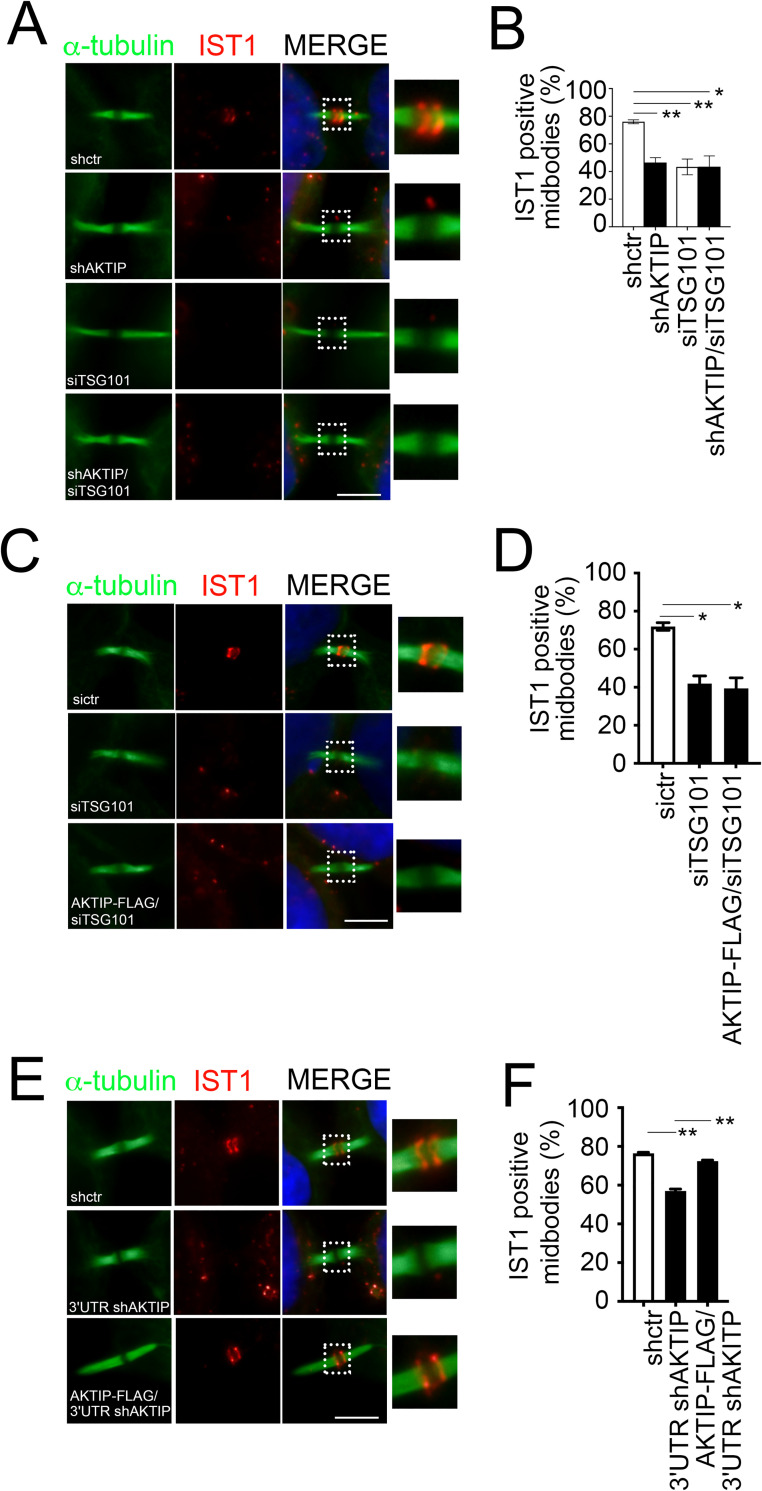
AKTIP and TSG101 act in a common pathway to promote IST1 recruitment. **(A-B)** Representative images of shctr and shAKTIP, siTSG101 and shAKTIP/siTSG101 HeLa cells stained for IST1 (red) and α-tubulin (green) and relative quantification. **(C-D)** Representative images of sictr, siTSG101 and AKTIP-FLAG/siTSG101 HeLa cells stained for IST1 (red) and α-tubulin (green) and relative quantification. **(E-F)** Representative images of shctr and 3’UTR shAKTIP, and AKTIP-FLAG/3’UTR shAKTIP HeLa cells stained for IST1 (red) and α-tubulin (green) and relative quantification. For results in (B, D and F) at least 100 midbodies per condition were counted. Scale bars, 5μm.

As a further step we looked at IST1 in shAKTIP, siTSG101 and shAKTIP/siTSG101 cells. We observe that both AKTIP and TSG101 are required for IST1 recruitment. Furthermore, we observe that the levels of the reduction of IST1 are comparable in shAKTIP, siTSG101 and shAKTIP/siTSG101 cells, suggesting that AKTIP and TSG101 act in a common path for the recruitment of IST1 to the midbody (Figs 9A and 9B and S7B and S7C).

To further explore whether both AKTIP and TSG101 were needed for IST1 recruitment to the midbody, we tested the ability of AKTIP to rescue TSG101 deficiency. To this end we created a stable AKTIP-FLAG overexpressing cell line. We verified the presence of AKTIP-FLAG at midbody by immunofluorescence with an anti-FLAG antibody and monitored the expression level of AKTIP in AKTIP-FLAG cells (S8A–S8C Fig). We next reduced the expression of TSG101 in AKTIP-FLAG cells by siTSG101 (S8D and S8E Fig). In this experimental setting, IST1 is not rescued by AKTIP overexpression (Fig 9C and 9D). To complete these data, we produced 3’-UTR shAKTIP and AKTIP-FLAG/3’-UTR shAKTIP cells. Here we can see that the IST1 defect observed in 3’-UTR shAKTIP cells is rescued in AKTIP-FLAG/3’-UTR shAKTIP cells (Figs 9E–9F and S8F).

Taken together, these results indicate that AKTIP and TSG101 cooperate in the recruitment of CHMP4B to the midbody, but act in two independent pathways. On the other hand, the absence of additivity and of rescue indicates, respectively, that AKTIP and TSG101 act in the same pathway on IST1 but are both needed for its recruitment to the midbody.

### AKTIP reduction causes defects of cytokinesis

**Fig 10:**
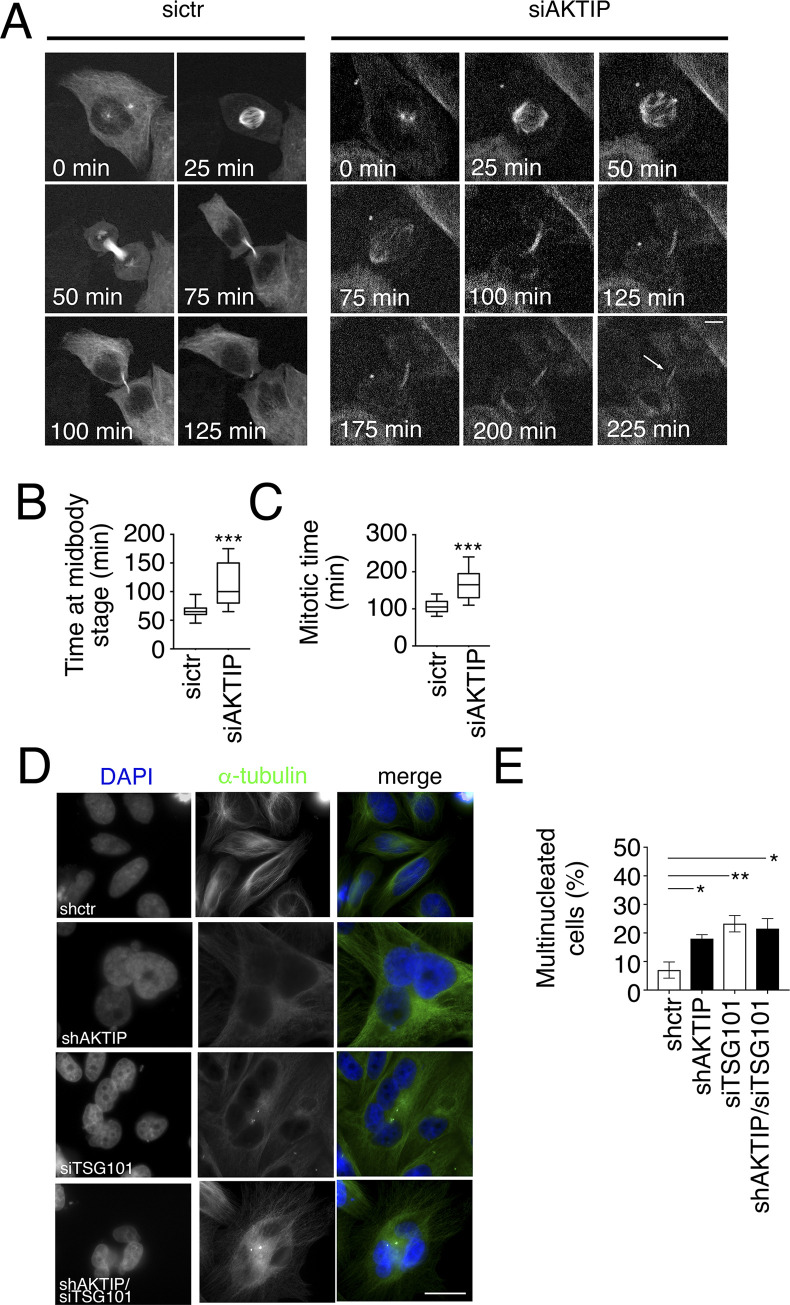
The reduction of AKTIP is associated with cytokinesis defects. **(A)** Selected frames from time-lapse microscopy of ctr or AKTIP depleted (siAKTIP) HeLa cells stably expressing mCherry-tubulin. The arrow points to an example of two cells that remain connected. Elapsed times are provided in each panel. **(B-C)** Quantitative analysis of time-lapse microscopy showing the time from prometaphase to abscission and from telophase to abscission. **(D-E)** Representative images and quantification of the percentage of multinucleated cells compared to normal interphase nuclei in shctr, shAKTIP, siTSG101, shAKTIP/siTSG101 HeLa cells. For results in (E) at least 200 nuclei per condition were counted. Scale bars, 5μm.

Given that AKTIP loss causes a reduction of IST1 and that IST1 functions in cytokinesis [38], we asked whether AKTIP would impact on cytokinesis. First, we explored whether AKTIP reduction had an impact on cell division by performing live cell microscopy using HeLa cells stably expressing mCherry tagged α-tubulin. This analysis shows that AKTIP reduction by siAKTIP (S9A Fig) causes cells to lengthen the abscission stage of cytokinesis from an average time of 107±5min to 169±12min (Fig 10A–10C and S6–S8 Movies). siAKTIP cells remain tethered together through their midbody for longer times with respect to control cells (Fig 10A–10C and S6–S8 Movies). Analyses on fixed cells confirm that a reduced AKTIP expression causes cytokinesis defects. Indeed, we observe that shAKTIP cells show an increase in the number of multinucleated cells (Fig 10D and 10E). As a further step, we analyzed multinucleation in siTSG101 and shAKTIP/siTSG101 cells. We observe that co-depletion generates multinucleated cell numbers comparable to those observed in cells depleted for AKTIP alone, further supporting that AKTIP and TSG101 act in the same pathway (Fig 10D and 10E).

Finally, since in our previous work we documented a role for AKTIP in telomere dysfunction [21], we next asked if the defects in cell division downstream to the reduced IST1 recruitment to the midbody could be due to telomeric dysfunction. To exclude this possibility, we analyzed AKTIP depleted cells and cells depleted of TRF2 by RNA interference (S9B Fig). TRF2 is a master element in the control of telomere integrity [39]. Immunofluorescence analysis of IST1 shows that while the reduction of AKTIP affects the recruitment of IST1 at the midbody, depletion of TRF2 does not (S9C and S9D Fig).

## Discussion

AKTIP is a protein with similarities with the ESCRT I subunit TSG101 [26,27]. Here we presented data that show the presence and structural organization of AKTIP at the midbody and provide evidence that AKTIP is a component of the ESCRT machinery operating at the midbody prior to the final constriction and severing stages.

**Fig 11:**
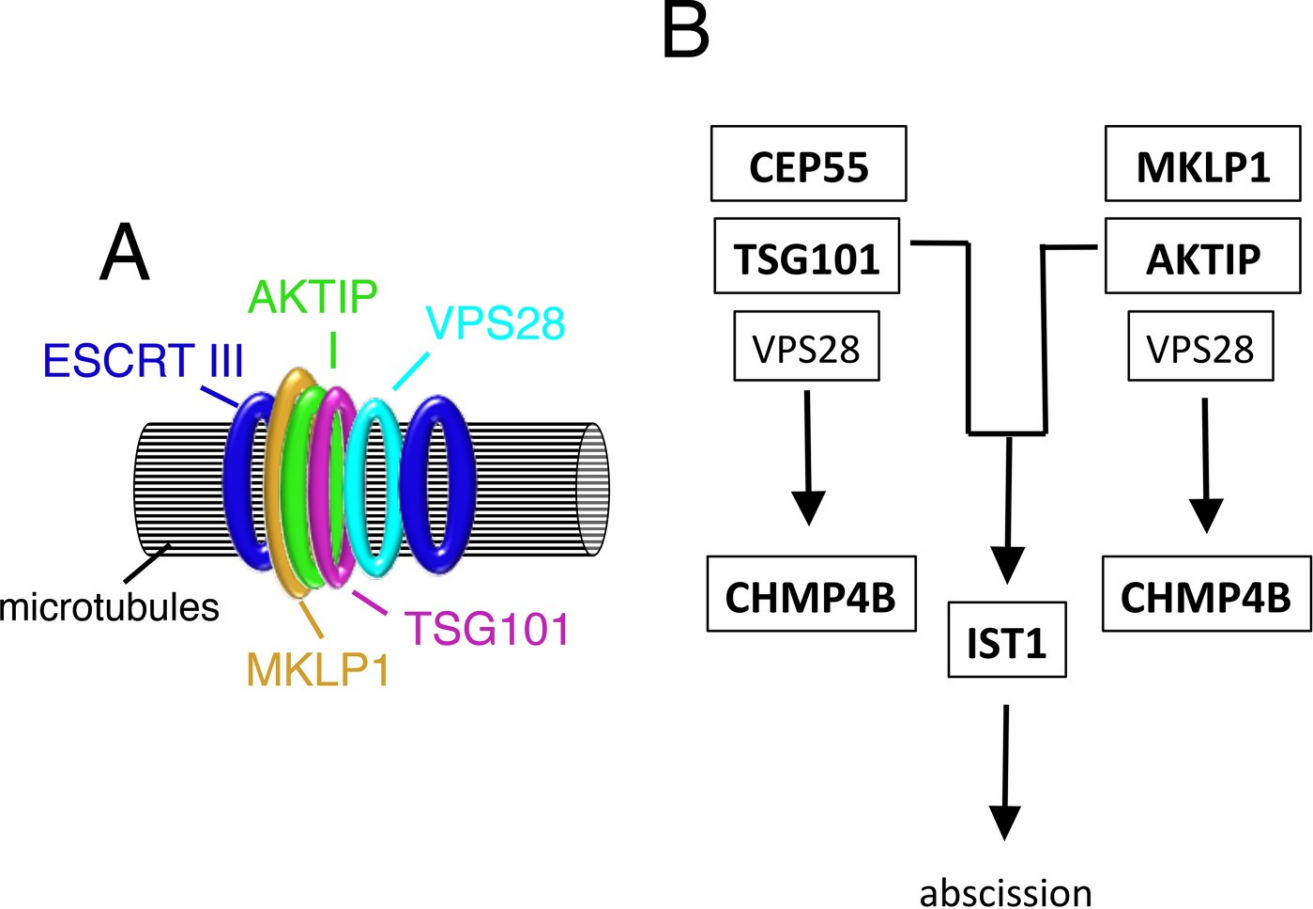
Schematic representation of AKTIP structural and functional association with the ESCRT machinery. **(A)** AKTIP localizes at the midbody in proximity with the annular structure formed by MKLP1 and with that formed by the ESCRT I subunit TSG101 and VPS28. AKTIP super-structure is flanked by the rings formed by the ESCRT III subunits CHMP4B, CHMP2A and IST1. **(B)** AKTIP is recruited to the midbody in a MKLP1 dependent, CEP55 independent way. AKTIP and TSG101 cooperate in the recruitment of CHMP4B to the midbody through independent routes, likely involving the ESCRT I subunit VPS28, that interacts with both TSG101 and AKTIP. AKTIP and TSG101 are needed for the recruitment of IST1 to the midbody, and act in a common pathway leading to abscission.

The first experimental evidence linking AKTIP to the ESCRT complex is in the characteristics of its supra-molecular organization. We show that AKTIP forms a large multimeric circular structure around microtubules at the midbody where the ESCRT complex is recruited [7,16]. The organization of AKTIP into a circular super-structure is like that of ESCRT and ESCRT associated factors. These factors form a series of disks and rings in the dark zone, the central area of the midbody, serving as a platform for the successive assembly and structural evolution of the ESCRT machinery. The assembly of AKTIP as a ring is preceded by a phase where AKTIP and α-tubulin have similar localizations. When the ESCRT elements have formed full circular structures, the AKTIP ring is found at the center, localizes in close proximity with MKLP1 and with the ESCRT I subunits TSG101 and VPS28, and in between the rings formed by the ESCRT III components IST1, CHMP2A and CHMP4B (Fig 11A).

A second set of experimental evidence links AKTIP to the ESCRT I module. AKTIP biochemically interacts with VPS28. VPS28 is an ESCRT I member required for the assembly of ESCRT complex [4,32]. VPS28 interacts with the ESCRT I TSG101 [6,31,32] and with the ESCRT II VPS36 [8,9]. AKTIP and TSG101 are both characterized by a UEV domain [26,27], however the two proteins differ significantly at both their N- and C-termini. This suggests that VPS28 interacts with AKTIP and TSG101 using different mechanisms. Indeed, we demonstrate that the interaction between AKTIP and VPS28 happens through the region including the UEV domain of AKTIP. The region of VPS28 implicated in binding to AKTIP is the N-terminal domain as it is the case for the binding to TSG101 [9]. AKTIP and TSG101 do not compete for their interaction with VPS28 and are concomitantly detected at the midbody and co-immunoprecipitated with VPS28. However, AKTIP and TSG101 are recruited independently to the midbody and VPS28 is not needed for the recruitment of AKTIP or viceversa. In accordance with the differences between TSG101 and AKTIP indicated by the sequence and by structural prediction, AKTIP does not interact with SEPT9.

Interestingly, the positioning of AKTIP at the midbody is not controlled by CEP55. Coherently with this experimental evidence, the sequence of AKTIP does not include the CEP55 binding residues 158–162 identified in TSG101 [40]. AKTIP recruitment is, on the other hand, dependent on the centralspindlin component MKLP1. Such a CEP55 independent, MKLP1 dependent recruitment has been reported for *Drosophila* [15] and in mouse primary fibroblasts [14].

The third piece of evidence functionally associating AKTIP with the ESCRT machinery is the impact of AKTIP on ESCRT III recruitment to the midbody. Notably, the combined depletion of AKTIP and TSG101 reduces the recruitment of the ESCRT III member CHMP4B. On the other hand, the single depletion is not sufficient to impair CHMP4B recruitment. These results show that AKTIP and TSG101 act in parallel arms on the recruitment of CHMP4B (Fig 11B). Previous work shows that the depletion of TSG101 alone is not sufficient to reduce the recruitment of CHMP4B, but TSG101 contributes to the process via VPS28 and its interaction with VPS36 [32]. The interaction of AKTIP with VPS28 suggests that AKTIP and TSG101 could both contribute to the recruitment of CHMP4B to the midbody via the VPS28/VPS36 route.

A direct relationship between members of the ESCRT I module and IST1 has been previously described and indicated as a path modulating a subset of the ATPase VPS4 activity at the midbody [38]. We report in this work a new link of the ESCRT I module with IST1. We show that either AKTIP or TSG101 depletion, or co-depletion of AKTIP and TSG101, reduces the levels of IST1 at the midbody. This axis is common for TSG101 and AKTIP, and thus presumably different from the route controlling CHMP4B involving TSG101 and AKTIP (Fig 11B). AKTIP and TSG101 act epistatically on the recruitment of IST1 but they are not interchangeable, since AKTIP overexpression does not rescue the IST1 defect caused by TSG101 reduction.

Given the function exerted by IST1 in the final stages of cell division [38], the AKTIP/TSG101/IST1 pathway is expected to be involved in cytokinesis. Indeed, we observe defects of cell division upon AKTIP depletion. Moreover, we observe the same degree of multinucleation defects in either TSG101, or AKTIP, or co-depleted cells. As for IST1, the absence of additivity in the data obtained upon single and double depletion suggests a common pathway in which both AKTIP and TSG101 play a role.

Collectively, the results presented demonstrate for the first time that AKTIP, a protein similar to the ESCRT I component TSG101, functionally associates with the ESCRT I module at the midbody and is required for the assembly of the ESCRT III module implicated in the final stages of cytokinesis. In future work, it will be interesting to explore if the AKTIP association with the ESCRT machinery has an impact on cancer, which has been indicated for TSG101 [41] and more recently reported for the ESCRT III subunit CHMP4C [42]. In addition, because of the enrichment of AKTIP at the nuclear periphery in interphase cells, it will be important to investigate if AKTIP also plays a role as part of the ESCRT machinery operating at the nuclear envelope. Interestingly, it is known that the ESCRT III subunit IST1, which we have shown here is affected by AKTIP reduction, plays a pivotal role in the orchestration of nuclear sealing processes after nuclear rupture and post-mitotically [43]. In this respect, it is tempting to speculate that the phenotype of telomere damage that we observed in AKTIP depleted cells [21] could be due to defects in the nuclear envelope sealing processes, which would be consistent with the phenotype of DNA damage observed in cells with reduced levels of the nuclear envelope ESCRT subunit CHMP7 [43].

## Materials and methods

### Cell culture, transfection and transduction

HeLa cells (ATCC CCL-2) and 293T cells (ATCC CRL-11268) were grown at 37°C; 5% CO_2_ in DMEM (Life Technologies) supplemented with 10% FBS (Life Technologies) and 50U/ml penicillin and streptomycin (Life Technologies). For transient RNA interference, cells were cultured in 6-well plates and were transfected with 20μM siRNA oligonucleotides using Lipofectamine 2000 (Life Technologies) following manufacturer’s instructions. Cells were collected or fixed 48hrs post-transfection. To obtain cell lines stably interfered for AKTIP or TRF2 we used lentivirus (LV) mediated interference and the LV-shAKTIP (shAKTIP), LV-shTRF2 (shTRF2) and LV-scramble (shctr) vectors were described previously [21]. The multiplicity of infection (moi) used was 5pg p24/cell. Transduction was performed in complete medium supplemented with 8μg/ml polybrene (Sigma). After viral addition, cells were centrifuged for 30min at 1800rpm at room temperature (RT), incubated for 3hrs at 37°C and then transferred to fresh complete medium. Seventy-two hrs post-infection, cells transduced with LVs were subjected to selection in complete medium supplemented with 2μg/ml puromycin (Sigma) and kept under these conditions for further analyses. For co-depletion experiments shAKTIP and shctr cells were transfected in 6-well plates with 20μM of the indicated siRNAs using Lipofectamine 2000 (Life Technologies) following manufacturer’s instructions. The following siRNAs were used: MISSION siRNA Universal Negative Control_#1_SIC001 as control; Sigma, SASI_Hs01_0086240 for AKTIP; Sigma, SASI_HS01_00102541 for MKLP1; Sigma, SASI_Hs01_0018067 for TSG101; Sigma, SASI_Hs01_00207653; Dharmacon SMARTpool, M-006893-01-0005, for CEP55. For the rescue experiments we obtained a cell line expressing AKTIP-FLAG, by the expression of the LV-AKTIP-FLAG obtained by replacing GFP pWPXLD (Addgene) with AKTIP-MYC-FLAG from pCMV6-Entry-AKTIP-FLAG plasmid (OriGene), and a control cell line transducing HeLa cells with an empty pWPXLD devoid of GFP. The moi used was 1pg p24/cell and the transduction protocol is described above. Then we downregulated endogenous AKTIP by the LV-mediated expression of sh 3’UTR shAKTIP described previously [21] at a moi of 1pg p24/cell following the protocol described above. As negative control we used LV-scramble (shctr) vectors described previously [21]. Seventy-two hrs post-infection, cells transduced with LVs were subjected to selection in complete medium supplemented with 2μg/ml puromycin (Sigma) and kept under these conditions for further analyses.

### Quantification of gene expression

One-week post-transduction, cells were lysed by addition of TRIzol reagent (Invitrogen) and RNA extracted according to the manufacturer’s instructions. After DNase treatment (Invitrogen), RNA was reverse transcribed into cDNA as already described [44]. Q-PCR reactions were carried out as previously described [45], using the following primers:

AKTIP Forward 5’- TCCACGCTTGGTGTTCGAT-3’;

AKTIP Reverse 5’-TCACCTGAGGTGGGATCAACT-3’;

TRF2 Forward 5’- TCCTCACGATGGCCAAAAAG-3’;

TRF2 Reverse 5’- GCTGTTTATCTTCCTTCCCTGTACT-3’;

GAPDH Forward 5’-TGGGCTACACTGAGCACCAG-3’;

GAPDH Reverse 5’-GGGTGTCGCTGTTGAAGTCA-3’

and analyzed with the 2^–ΔΔCq^ method as previously described [46]. For Western blotting, one week post-transduction with LV-shAKTIP or LV-shctr and 72hrs post-transfection with siRNAs, protein extracts were obtained as previously described [21] and quantified by Bradford assay. 100μg protein extracts were loaded onto pre-cast 4–12% gradient acrylamide gels (Novex, Life Technology). After electro-blotting filters were incubated with anti-AKTIP (HPA041794 Sigma) and anti-actin-HRP conjugated (sc-1615, Santa Cruz Biotechnology) antibodies. Filters were then incubated with anti rabbit HRP-conjugated secondary antibody (sc-2357, Santa Cruz Biotechnology). Detection was performed using the enhanced chemiluminescence system (Clarity ECL, Biorad).

### Immunofluorescence and microscopy

HeLa cells were seeded onto glass coverslips in 6-well plates and fixed with 3.7% formaldehyde in PBS for 10min. Cells were then permeabilized with 0.25% Triton X-100 in PBS for 5min, treated with PBS 1% BSA for 30min and then stained with primary antibodies in PBS 1% BSA for 1hr at RT. For AKTIP-TSG101 co-immunofluorescence, HeLa cells were seeded onto glass coverslips, fixed as previously described [7], permeabilized in PBS- 0.1% Triton X-100 for 2hrs and successively blocked as described above. The following primary antibodies were used: anti-AKTIP (WH0064400M2 clone 2A11 and HPA041794 Sigma), anti-Tubulin [YL1/2] Rat monoclonal (Abcam, ab6160), anti-CHMP4B (Proteintech, 13683-1-AP), anti-IST1 (Proteintech, 51002-1-AP), anti-CHMP2A (Proteintech, 10477-1-AP), anti-CEP55 (Abnova, H00055165-A01) anti-MKLP1 (SantaCruz Biotechnology, sc-22793) anti-ALIX (SantaCruz Biotechnology, sc-53540) anti-TSG101 (SantaCruz Biotechnology, sc-7964), anti-FLAG (add Sigma, F3165) and anti VPS28 (Proteintech 15478-1-AP). Alexa488, Alexa568, Alexa647 or FITC conjugated secondary antibodies were diluted in PBS and inclubated for 45min at RT. Nuclei were visualized using DAPI (4,6 diamidino-2-phenylindole) and coverslips were mounted in Vectashield H-1000. Slides were imaged using Zeiss AxioImager Z1 (EBL) equipped with an Axiocam 506 color. Confocal laser scanning microscopy was performed with Corrsight confocal scanning microscope. Greyscale images were pseudocoloured and combined in Adobe Photoshop CC to create merged images.

For Structured illumination microscopy (3D-SIM) and Multispot-SIM (MSIM) imaging, HeLa cells were seeded onto glass coverslips (high performance coverslips #1.5H, #0107052—Marienfeld superior) in 6-well plates and fixed with 3.7% formaldehyde in PBS for 10min at RT and then incubated in 50mM NH4Cl/PBS (15min). Cells were incubated with primary and secondary antibodies diluted in PBS-BSA 1% for 1hr at RT and washed in PBS. Acquisition of 3D-SIM images was performed using a DeltaVision OMX v4 Blaze microscope (GE Healthcare) equipped with the BGR-FR filter drawer for acquisition of 3D-SIM images, an Olympus Plan Apochromat 100×/1.4 PSF oil immersion objective lens and liquid-cooled Photometrics Evolve EM-CCD cameras for each channel. 15 images per section per channel were acquired with a z-spacing of 0.125μm [47,48]. Structured illumination reconstruction and wavelength alignment were done post-acquisition using the SoftWorX software (GE Healthcare). Acquisition of MSIM images was performed through a Nikon Eclipse Ti equipped with: X-Light V2 spinning disk combined with a VCS (Video Confocal Super resolution) module (CrestOptics) based on structured illumination. LDI laser source (89 North) and Prime BSI Scientific CMOS (sCMOS) camera with 6.5μm pixels (Photometrics) were used. The images were acquired by using Metamorph software version 7.10.2. (Molecular Devices) with a Nikon 100x/1.45 Plan Apochromat Lambda oil immersion objective at a z-spacing of 0.1μm. In order to achieve super-resolution, raw data obtained by the VCS module were processed with a modified version of the joint Richardson-Lucy (jRL) algorithm [49], where the out of focus contribution of the signal has been explicitly added in the image formation model used in the jRL algorithm, and evaluated as a pixel-wise linear "scaled subtraction” of the raw signal [50].

3D volume reconstructions and movies generation were done in Imaris (Bitplane, Oxford Instruments). Image analysis and quantification was performed using Image J/FIJI [51], Excel (Microsoft) and Prism (Graphpad) software. Fluorescence intensity values represent the average fluorescence intensity measured from a 2.7μm wide band along the axis of the tubulin bundle.

### Yeast two-hybrid assays

Yeast two hybrid assays were performed as previously described [52]. Briefly, yeast Y190 cells were co-transformed with plasmids encoding the indicated proteins fused to the VP16 activation domain (pHB18) and AKTIP fused to the Gal4 DNA-binding domain (pGBKT7). Co-transformants were selected on SD-Leu-Trp agar for 72hrs at 30°C, harvested, and LacZ activity was measured using a liquid β-galactosidase assay employing chlorophenolred-ß-D-galactopyranoside (Roche) as a substrate.

### GST pull down and GFP-TRAP

GST pull down were performed as previously described [53]. Full length VPS28, NT (1–120 aa) VPS28, CT (121–221 aa) VPS28, and IST1 were cloned as a GST-fusion into pCAGGS/GST. 293T cells were co-transfected with 1μg per well of 6-well plate of either pCAGGS/GST or pCAGGS/GST fused proteins and with 1μg per well of 6-well plate of pCMV6-Entry-AKTIP-Myc-Flag (ORIGENE) or with 1μg per well of 6-well plate of HA-AKTIP (pCR3.1) or with N-terminal GFP tagged full length AKTIP, NT (1–76 aa) AKTIP, UEV (78–220 aa) AKTIP, CT (221–292 aa) AKTIP (pNG72-GFP) for 48hrs. To analyze AKTIP, VPS28, TSG101 mutual interaction, 293T cells were co-transfected with 1μg per well of 6-well plate of either pCAGGS/GST or pCAGGS/GST-VPS28 with 0.5μg per well of 6-well plate of N-terminal GFP tagged TSG101 (pNG72-GFP) and increasing amounts (0, 50, 100, 500 or 1000ng) of full length HA-AKTIP (pCR3.1). Cells were then harvested and lysed in NP40 lysis buffer (150mM NaCl, 50mM Tris pH7.5, 1mM EDTA). Clarified lysates were incubated with glutathione-Sepharose beads (Amersham Biosciences) for 3hrs at 4°C and washed three times with wash buffer (50mM Tris-HCl, pH 7.4, 150mM NaCl, 5mM EDTA, 5% glycerol, 0.1% Triton X-100). Bead-bound proteins were eluted by boiling in 100μl of Laemmli sample buffer and resolved by SDS-PAGE, as previously described [52].

For GFP-TRAP 293T cells were transfected with plasmids encoding GFP-tagged versions of ESCRT-subunits or SEPT9 isoforms and pCMV6-Entry-AKTIP-Myc-Flag (ORIGENE). pEGFPC2 plasmids encoding SEPT9 isoforms were kind gifts from Ulrike Eggert (Kings College, London). Cells were then lysed in pull down lysis buffer (150mM NaCl, 50 mM Tris pH7.5, 1 mM EDTA, 1% glycerol, 1% NP40, protease inhibitors), cleared by centrifugation and incubated magnetic agarose GFP-trap beads (Chromotek) for 10 minutes. Captured fraction separated by magnetisation and washed with pull down wash buffer (150mM NaCl, 50 mM Tris pH7.5, 1 mM EDTA, 1% glycerol, 0.1% NP40). Eluted proteins were recovered with 2x LDS sample buffer and resolved by SDS- PAGE. Resolved proteins were transferred onto nitrocellulose by Western blotting and were probed with the indicated antibodies in 5% milk. HRP-conjugated secondary antibodies were incubated with ECL Prime enhanced chemiluminescent substrate (GE Healthcare) and visualized by exposure to autoradiography film. The following primary antibodies were used: anti-HA (ABIN100176, Antibodies Online), anti-MYC (sc-789, Santa Cruz Biotechnology), anti-GST (10000–0 AP, Proteintech), anti-GFP (Roche, 11814460001). The secondary antibodies used were goat anti-rabbit HRP-conjugated (Cell Signaling).

### Bioinformatics

AKTIP and TSG101 sequence alignment was performed with Jalview (ClustalO alignment with default settings). The PDB AKTIP homology model [21] and the X-ray structure of TSG101 [35] were used for superposition, molecular visualization and analysis performed with UCSF Chimera 1.13.1 [54]

### Statistics

Statistical analyses were performed using Excel and Graphpad Prism software. Results are shown as mean ± SEM of at least three independent experiments. Data were analyzed using unpaired two-tailed Student’s t-test. p-values below 0.05 were considered significant and reported in figures as *p<0.05; **p<0.01; *** p<0.001. p values above 0.05 were considered not significant and were not reported in figures.

## Supporting information

S1 Fig Specificity of the signal of AKTIP at the midbody and production of cells with stable knock down of AKTIP. **(A)** AKTIP localization at the midbody in HeLa cells using anti-AKTIP antibodies WH0064400M2 clone 2A11 and HPA041794. **(B)** Detection of exogenous AKTIP-FLAG in transfected cells using anti-FLAG antibody. **(C)** Immunofluorescence of HeLa cells transduced with a control lentivector containing a scramble interfering sequence (shctr) or with AKTIP interfering sequence (shAKTIP) stained for α-tubulin (green) and AKTIP (red). Scale bars, 5μm. **(D)** Western blotting of AKTIP expression in shAKTIP and shctr cells. Actin was used as loading control. **(E)** Representative Q-PCR of AKTIP mRNA expression in shAKTIP and shctr cells.
(TIF)

S2 Fig AKTIP interaction assay with VPS28. Western blotting showing that AKTIP interacts with GST-VPS28 but not with GST alone. Cells were transfected with plasmids encoding the indicated fusion proteins. Purified VPS28-GST or GST alone were used to pull down interacting proteins; cell lysates and glutathione-bound fractions were then analyzed with HA antisera. GST-pull downs were repeated two times. In the HA-AKTIP blot we observe a major band at the correct molecular weight, in the pulldown fractions we observe also degradation products that retain the GST moiety.
(TIF)

S3 Fig AKTIP-TSG101 sequence alignment and AKTIP interaction assay with ESCRTII. **(A)** Grey squares: identical residues in AKTIP and TSG101 sequences. Blue boxes: H1, H2, H3, and H4 TSG101 helices. Red boxes: H2, H3, H4, H5 and H6 helices predicted in AKTIP model. White arrows: β-strands. **(B)** Western blotting showing that AKTIP does not interact with VPS25, VPS22 and VPS36 and confirming its interaction with VPS28. Cells were transfected with the indicated fusion proteins. Purified VPS25-GFP, VPS22-GFP, VPS36-GFP, VPS28-GFP or GFP alone were used to trap interacting proteins; then cell lysates and GFP-trapped fractions were analyzed with MYC antisera. GFP-TRAP were repeated twice.
(TIF)

S4 Fig Production of CEP55 depleted HeLa cells. **(A-B)** Representative images and quantification of sictr and siCEP55 transiently transfected HeLa cells stained for CEP55 (red) and α-tubulin (green). Scale bar, 2.5μm. For results in (B) at least 100 midbodies per condition were counted. **(C-D)** Representative images and quantification of multinucleated cells in sictr and siCEP55 HeLa cells. For results in (D) at least 200 nuclei per condition were counted. Scale bar, 5μm.
(TIF)

S5 Fig Production and functional characterization of ALIX and VPS28 depleted HeLa cells. **(A-B)** Representative images and quantification of sictr and siTSG101 transiently transfected HeLa cells stained for TSG101 (red) and α-tubulin (green). **(C-D)** Representative images and relative quantification of shctr and shAKTIP HeLa cells stained for ALIX (red) and α-tubulin (green) showing that ALIX is present at the midbody in shAKTIP cells. **(E-F)** Representative images and relative quantification of sictr and siALIX HeLa cells stained for AKTIP (red) and α-tubulin (green) showing that AKTIP is present at the midbody in siALIX cells. **(G-H)** Representative images and quantification of sictr and siALIX transiently transfected HeLa cells stained for ALIX (red) and α-tubulin (green). **(I-J)** Representative images and quantification of sictr and siVPS28 transiently transfected HeLa cells stained for VPS28 (red) and α-tubulin (green). Scale bars, 5μm. For results in (B, D, F, H and J) at least 80 midbodies per condition were counted.
(TIF)

S6 Fig Production of MKLP1 depleted HeLa cells. **(A-B)** Representative images and quantification of sictr and siMKLP1 transiently transfected HeLa cells stained for α-tubulin (green) and MKLP1 (red). For results in (B) at least 25 midbodies per condition were counted. Scale bar, 2.5μm.
(TIF)

S7 Fig CHMP4B analysis and production of shAKTIP/siTSG101, shAKTIP/siALIX HeLa cells. **(A)** Quantification of the percentage of midbodies with abnormally localized CHMP4B (black) or with two rings (red) and with cones or spirals (white) of CHMP4B referred to CHMP4B positive midbodies in shctr and shAKTIP HeLa cells stained for CHMP4B and α-tubulin (Fig 8A and 8C). **(B-C)** Representative images and relative quantification of shctr, shAKTIP, siTSG101, and shAKTIP/siTSG101 HeLa cells, stained for α-tubulin (green) and TSG101 (red). **(D-E)** Representative images and relative quantification of shctr, shAKTIP, siALIX, and shAKTIP/ siALIX HeLa cells, stained for α-tubulin (green) and ALIX (red). For results in (C and E) at least 100 midbodies per condition were counted. Scale bars, 2.5μm.
(TIF)

S8 Fig Production of AKTIP-FLAG, AKTIP-FLAG/3’UTR shAKTIP and AKTIP-FLAG/siTSG101 HeLa cells. **(A-B)** Representative images and relative quantification of ctr, and AKTIP-FLAG HeLa cells, stained for α-tubulin (green) and FLAG (red). **(C)** Representative Q-PCR of AKTIP mRNA expression in ctr and AKTIP-FLAG HeLa cells. **(D-E)** Representative images and relative quantification of sictr, siTSG101, AKTIP-FLAG/siTSG101 HeLa cells, stained for α-tubulin (green) and TSG101 (red). **(F)** Representative Q-PCR of AKTIP mRNA expression in shctr, 3’UTR shAKTIP and AKTIP-FLAG/3’UTR shAKTIP HeLa cells. For results in (B and E) at least 80 midbodies per condition were counted Scale bars, 2.5μm.
(TIF)

S9 Fig Depletion of AKTIP by siRNA and IST1 midbody recruitment analysis in shTRF2 HeLa cells. **(A)** Western blotting of AKTIP expression in siAKTIP and sictr cells. Actin was used as loading control. **(B)** Representative Q-PCR of TRF2 mRNA expression in shTRF2 and shctr HeLa cells. **(C-D)** Representative images and relative quantification of shctr, shAKTIP, and shTRF2 HeLa cells, stained for α-tubulin (green) and IST1 (red). For results in (D) at least 100 midbodies per condition were counted. Scale bar, 2.5μm.
(TIF)

S1 Movie AKTIP localization at mid stage midbody. 3D volume rendering of mid stage midbody imaged with 3D-SIM. HeLa cells were stained with antibodies against α-tubulin (green) and AKTIP (red). The volume rendering and the movie generation were performed with IMARIS software (Bitplane, Oxford Instruments).
(MP4)

S2 Movie AKTIP localization at late stage midbody. 3D volume rendering of late stage midbody imaged with 3D-SIM. HeLa cells were stained with antibodies against α-tubulin (green) and AKTIP (red). The volume rendering and the movie generation were performed with IMARIS software (Bitplane, Oxford Instruments).
(MP4)

S3 Movie AKTIP and TSG101 localization at the midbody. 3D volume rendering of midbody imaged with MSIM. HeLa cells were stained with antibodies against AKTIP (green), and TSG101 (red). The volume rendering and the movie generation were performed with IMARIS software (Bitplane, Oxford Instruments).
(MP4)

S4 Movie AKTIP and IST1 localization at mid stage midbody. 3D volume rendering of mid stage midbody imaged with 3D-SIM. HeLa cells were stained with antibodies against α-tubulin (green), IST1 (blue) and AKTIP (red). The volume rendering and the movie generation were performed with IMARIS software (Bitplane, Oxford Instruments).
(MP4)

S5 Movie AKTIP and IST1 localization at late stage midbody. 3D volume rendering of late stage midbody imaged with 3D-SIM. HeLa cells were stained with antibodies against α-tubulin (green), IST1 (blue) and AKTIP (red). The volume rendering and the movie generation were performed with IMARIS software (Bitplane, Oxford Instruments).
(MP4)

S6 Movie Time-lapse of AKTIP depleted cells. Time-lapse microscopy of HeLa cells stably expressing mCherry-tubulin treated with siAKTIP. The movie generation was performed with Fiji software.
(AVI)

S7 Movie Time-lapse of AKTIP depleted cells. Time-lapse microscopy of HeLa cells stably expressing mCherry-tubulin treated with siAKTIP. The movie generation was performed with Fiji software.
(AVI)

S8 Movie Time-lapse of control treated cells. Time-lapse microscopy of HeLa cells stably expressing mCherry-tubulin treated with ctr. The movie generation was performed with Fiji software.
(AVI)
