# Supplementary figures and images for: AKTIP interacts with ESCRT I and is needed for the recruitment of ESCRT III subunits to the midbody
Source: PLoS Genet. 2021 Aug 27;17(8):e1009757. doi: 10.1371/journal.pgen.1009757 (PMC8428793; doi:10.1371/journal.pgen.1009757)

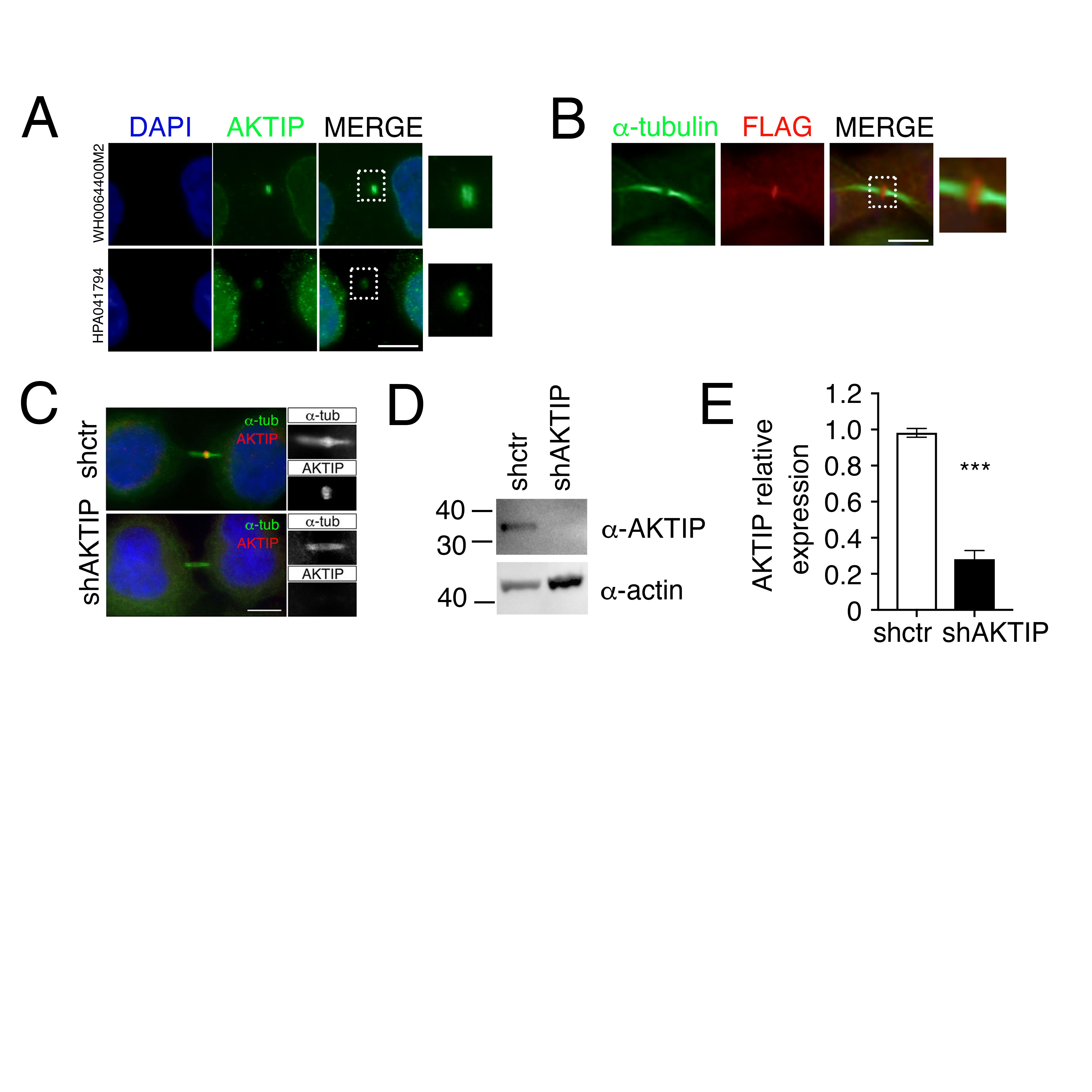

Supplement: S1 Fig — (A) AKTIP localization at the midbody in HeLa cells using anti-AKTIP antibodies WH0064400M2 clone 2A11 and HPA041794. (B) Detection of exogenous AKTIP-FLAG in transfected cells using anti-FLAG antibody. (C) Immunofluorescence of HeLa cells transduced with a control lentivector containing a scramble interfering sequence (shctr) or with AKTIP interfering sequence (shAKTIP) stained for α-tubulin (green) and AKTIP (red). Scale bars, 5μm. (D) Western blotting of AKTIP expression in shAKTIP and shctr cells. Actin was used as loading control. (E) Representative Q-PCR of AKTIP mRNA expression in shAKTIP and shctr cells. (TIF) [file pgen.1009757.s001.tif]

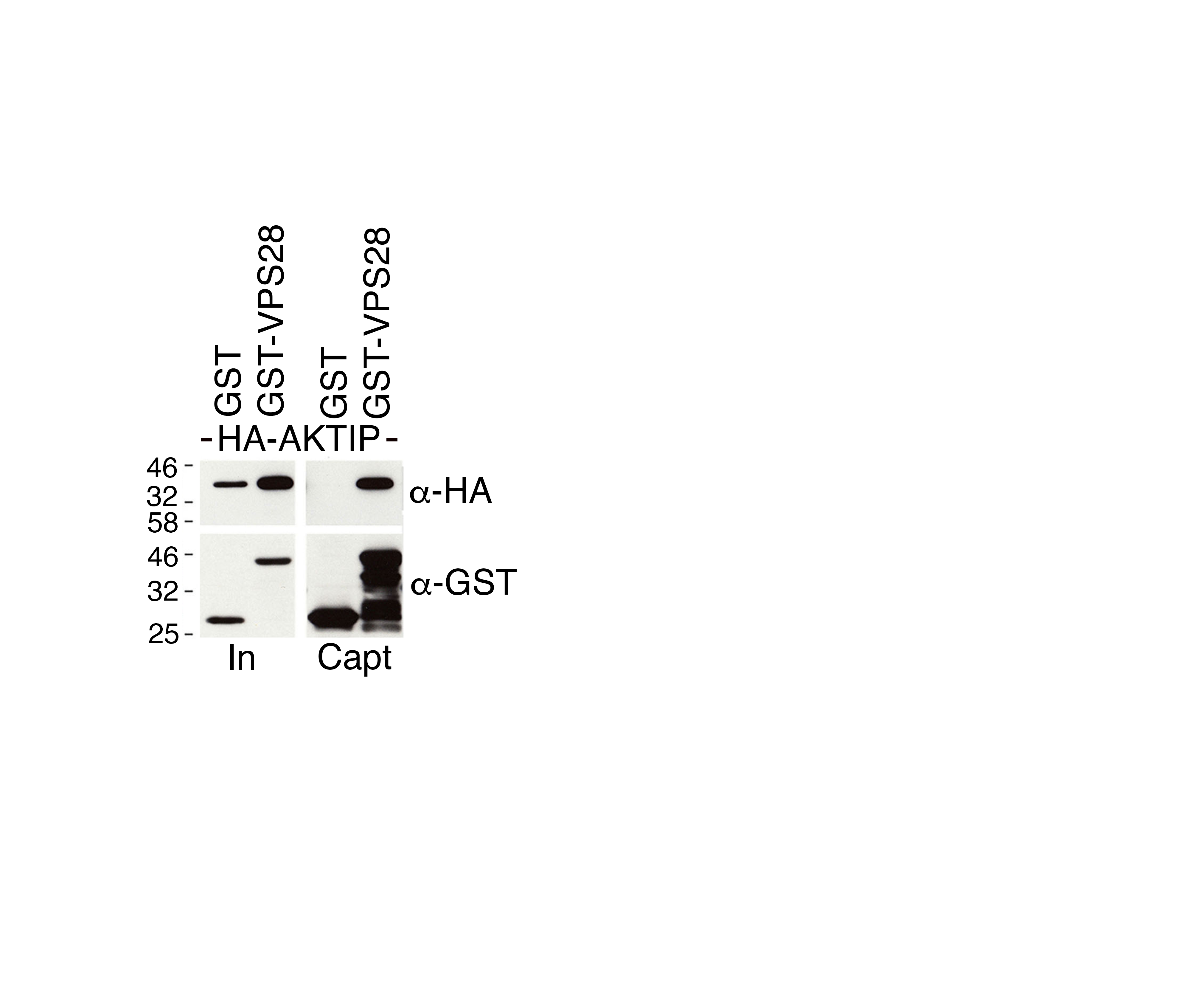

Supplement: S2 Fig — Western blotting showing that AKTIP interacts with GST-VPS28 but not with GST alone. Cells were transfected with plasmids encoding the indicated fusion proteins. Purified VPS28-GST or GST alone were used to pull down interacting proteins; cell lysates and glutathione-bound fractions were then analyzed with HA antisera. GST-pull downs were repeated two times. In the HA-AKTIP blot we observe a major band at the correct molecular weight, in the pulldown fractions we observe also degradation products that retain the GST moiety. (TIF) [file pgen.1009757.s002.tif]

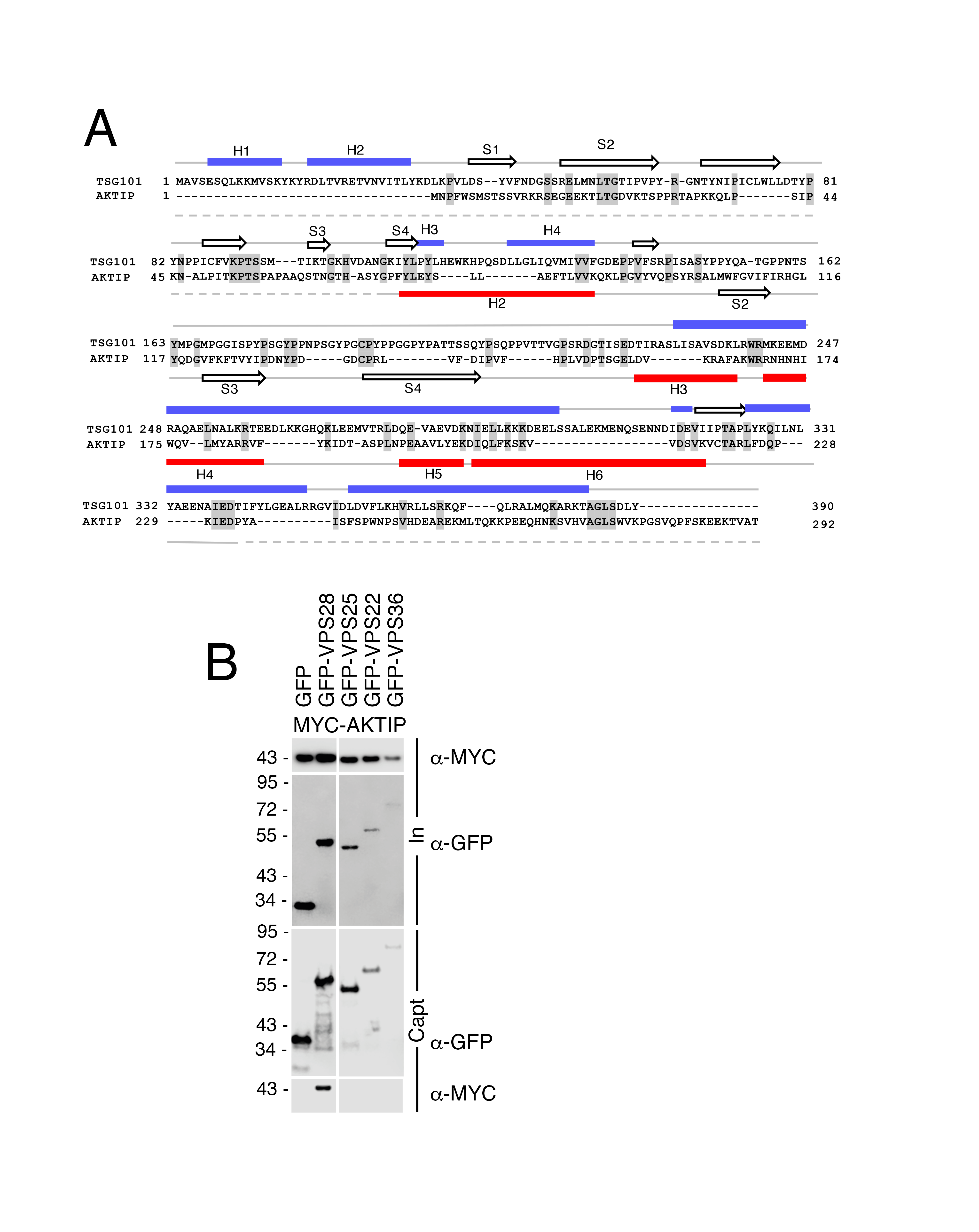

Supplement: S3 Fig — (A) Grey squares: identical residues in AKTIP and TSG101 sequences. Blue boxes: H1, H2, H3, and H4 TSG101 helices. Red boxes: H2, H3, H4, H5 and H6 helices predicted in AKTIP model. White arrows: β-strands. (B) Western blotting showing that AKTIP does not interact with VPS25, VPS22 and VPS36 and confirming its interaction with VPS28. Cells were transfected with the indicated fusion proteins. Purified VPS25-GFP, VPS22-GFP, VPS36-GFP, VPS28-GFP or GFP alone were used to trap interacting proteins; then cell lysates and GFP-trapped fractions were analyzed with MYC antisera. GFP-TRAP were repeated twice. (TIF) [file pgen.1009757.s003.tif]

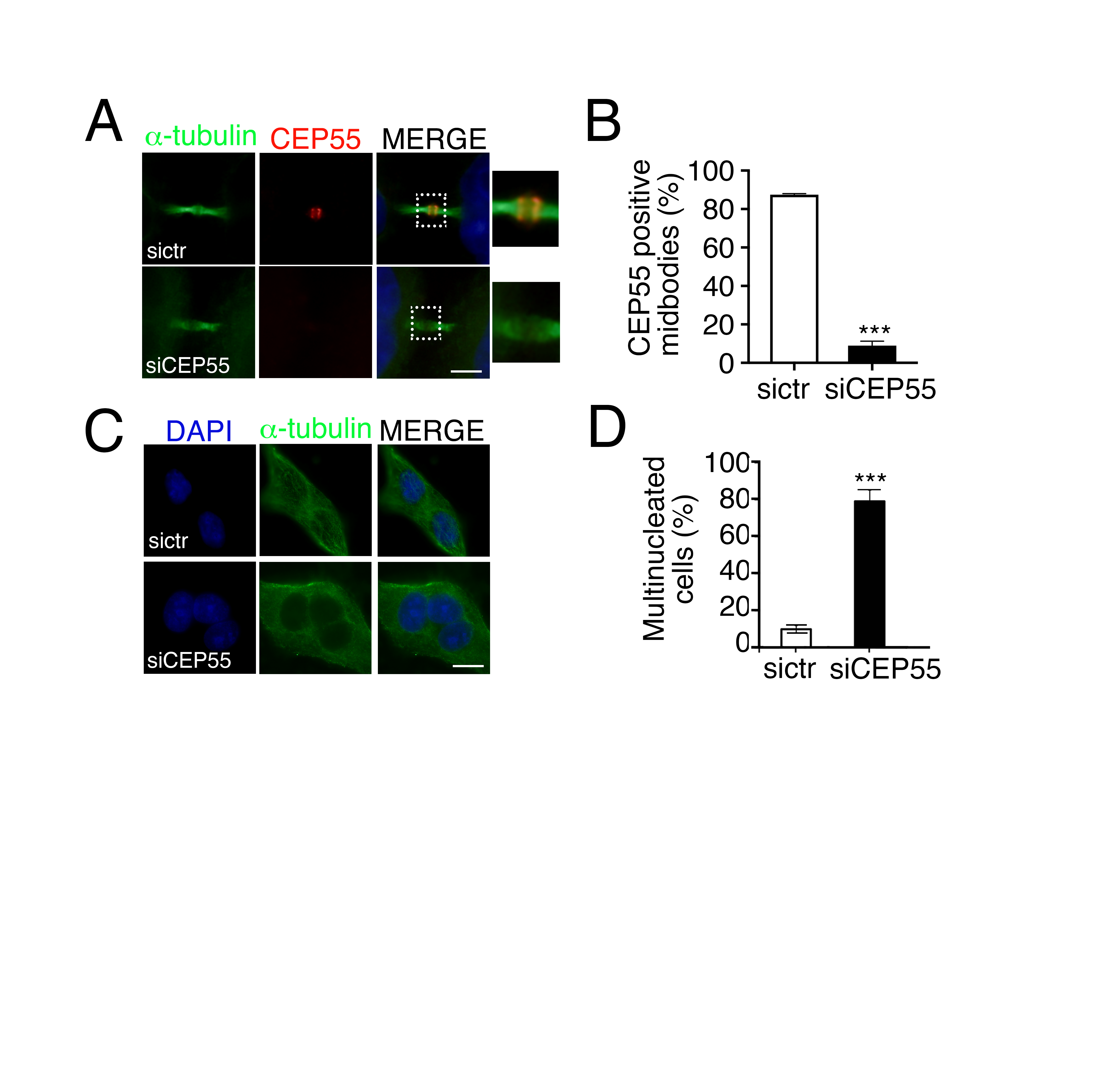

Supplement: S4 Fig — (A-B) Representative images and quantification of sictr and siCEP55 transiently transfected HeLa cells stained for CEP55 (red) and α-tubulin (green). Scale bar, 2.5μm. For results in (B) at least 100 midbodies per condition were counted. (C-D) Representative images and quantification of multinucleated cells in sictr and siCEP55 HeLa cells. For results in (D) at least 200 nuclei per condition were counted. Scale bar, 5μm. (TIF) [file pgen.1009757.s004.tif]

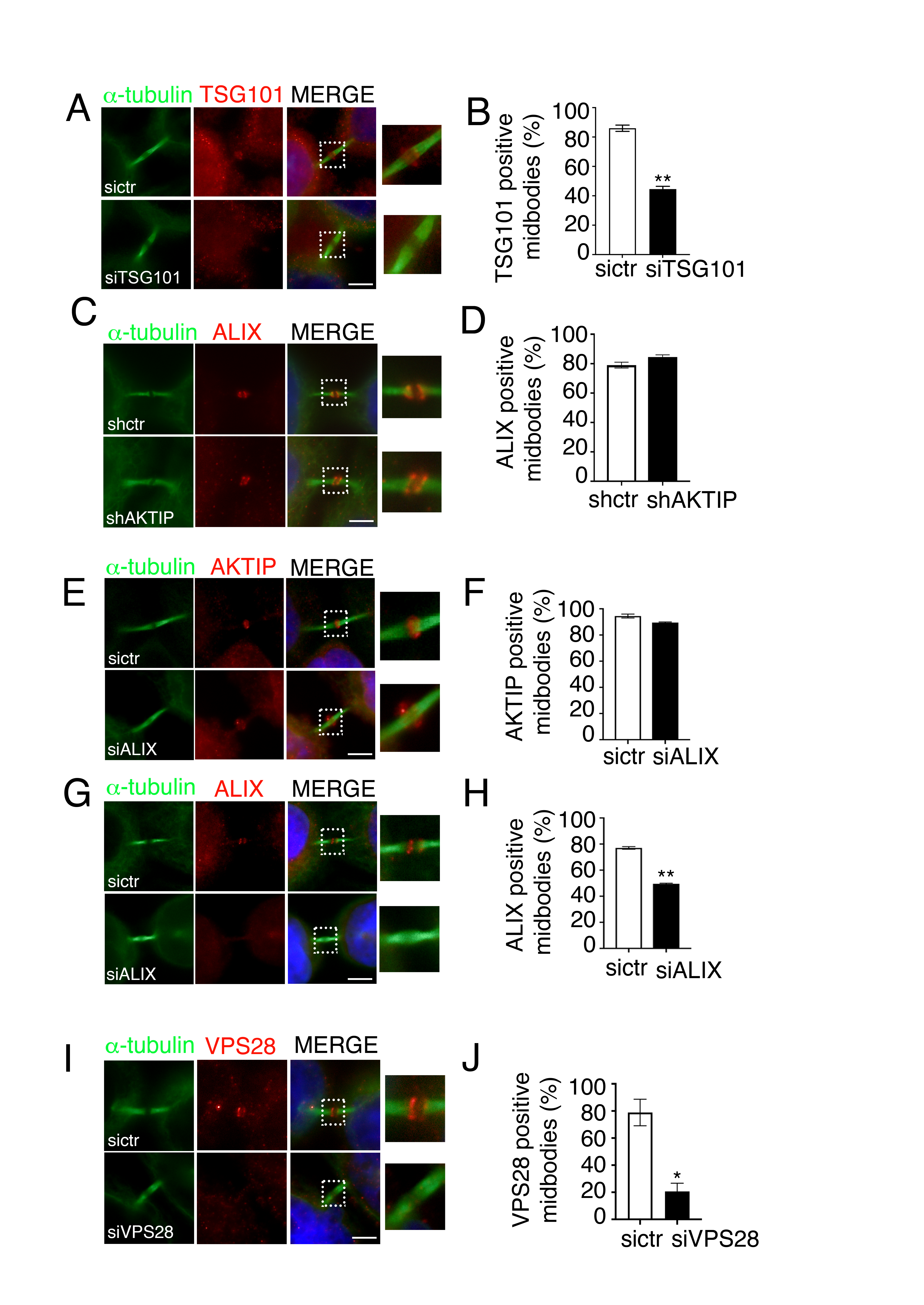

Supplement: S5 Fig — (A-B) Representative images and quantification of sictr and siTSG101 transiently transfected HeLa cells stained for TSG101 (red) and α-tubulin (green). (C-D) Representative images and relative quantification of shctr and shAKTIP HeLa cells stained for ALIX (red) and α-tubulin (green) showing that ALIX is present at the midbody in shAKTIP cells. (E-F) Representative images and relative quantification of sictr and siALIX HeLa cells stained for AKTIP (red) and α-tubulin (green) showing that AKTIP is present at the midbody in siALIX cells. (G-H) Representative images and quantification of sictr and siALIX transiently transfected HeLa cells stained for ALIX (red) and α-tubulin (green). (I-J) Representative images and quantification of sictr and siVPS28 transiently transfected HeLa cells stained for VPS28 (red) and α-tubulin (green). Scale bars, 5μm. For results in (B, D, F, H and J) at least 80 midbodies per condition were counted. (TIF) [file pgen.1009757.s005.tif]

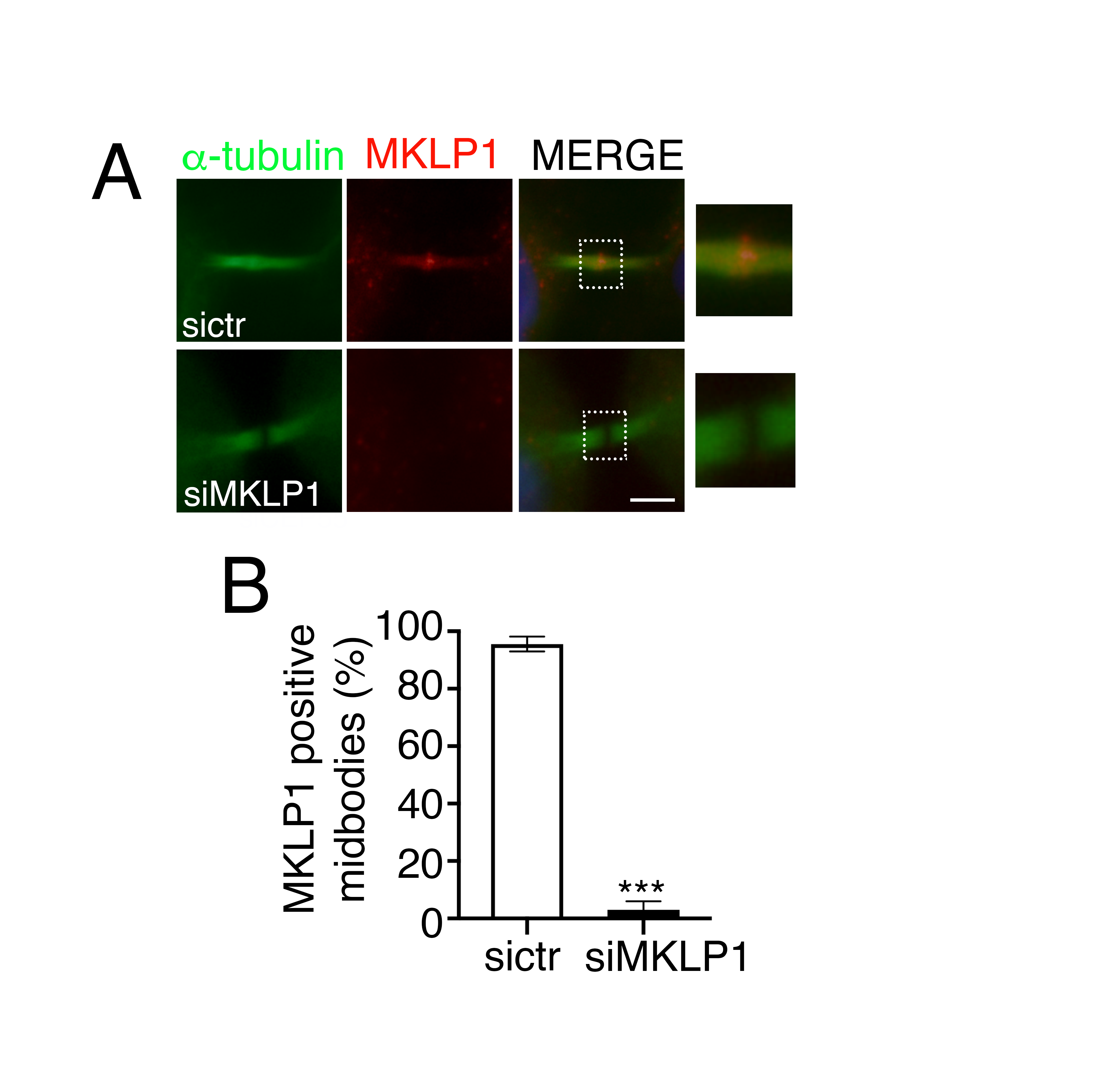

Supplement: S6 Fig — (A-B) Representative images and quantification of sictr and siMKLP1 transiently transfected HeLa cells stained for α-tubulin (green) and MKLP1 (red). For results in (B) at least 25 midbodies per condition were counted. Scale bar, 2.5μm. (TIF) [file pgen.1009757.s006.tif]

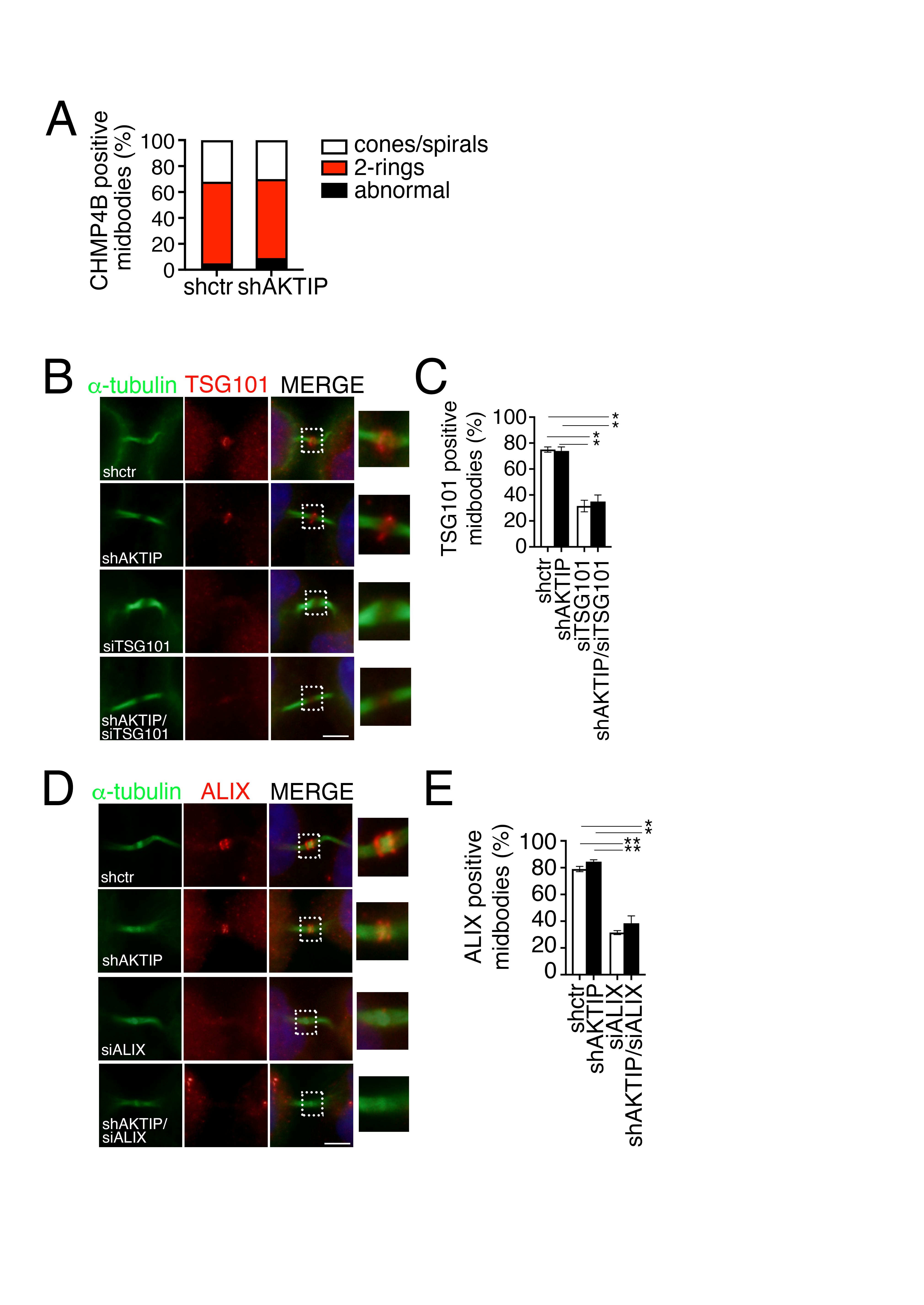

Supplement: S7 Fig — (A) Quantification of the percentage of midbodies with abnormally localized CHMP4B (black) or with two rings (red) and with cones or spirals (white) of CHMP4B referred to CHMP4B positive midbodies in shctr and shAKTIP HeLa cells stained for CHMP4B and α-tubulin (Fig 8A and 8C). (B-C) Representative images and relative quantification of shctr, shAKTIP, siTSG101, and shAKTIP/siTSG101 HeLa cells, stained for α-tubulin (green) and TSG101 (red). (D-E) Representative images and relative quantification of shctr, shAKTIP, siALIX, and shAKTIP/ siALIX HeLa cells, stained for α-tubulin (green) and ALIX (red). For results in (C and E) at least 100 midbodies per condition were counted. Scale bars, 2.5μm. (TIF) [file pgen.1009757.s007.tif]

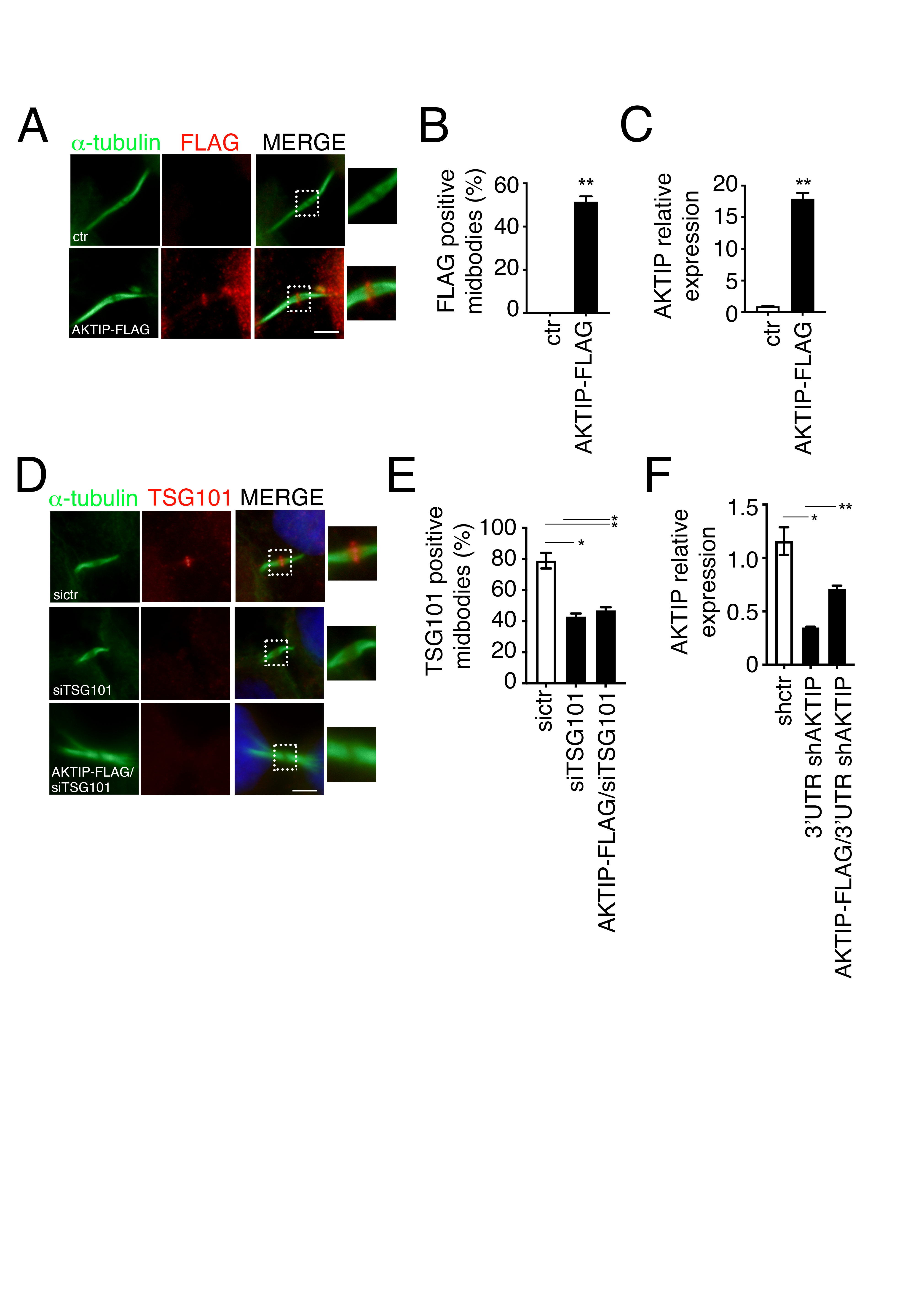

Supplement: S8 Fig — (A-B) Representative images and relative quantification of ctr, and AKTIP-FLAG HeLa cells, stained for α-tubulin (green) and FLAG (red). (C) Representative Q-PCR of AKTIP mRNA expression in ctr and AKTIP-FLAG HeLa cells. (D-E) Representative images and relative quantification of sictr, siTSG101, AKTIP-FLAG/siTSG101 HeLa cells, stained for α-tubulin (green) and TSG101 (red). (F) Representative Q-PCR of AKTIP mRNA expression in shctr, 3’UTR shAKTIP and AKTIP-FLAG/3’UTR shAKTIP HeLa cells. For results in (B and E) at least 80 midbodies per condition were counted Scale bars, 2.5μm. (TIF) [file pgen.1009757.s008.tif]

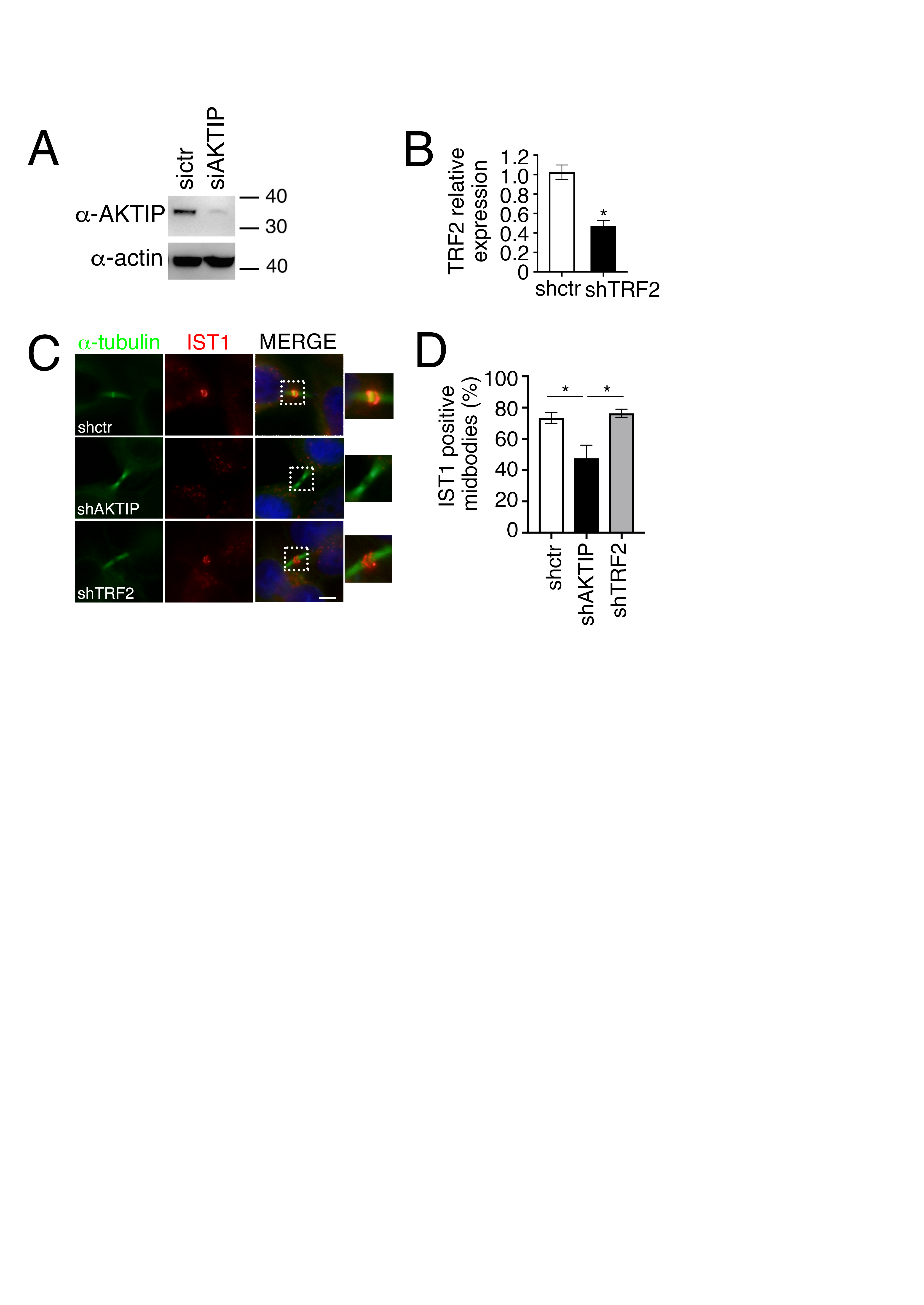

Supplement: S9 Fig — (A) Western blotting of AKTIP expression in siAKTIP and sictr cells. Actin was used as loading control. (B) Representative Q-PCR of TRF2 mRNA expression in shTRF2 and shctr HeLa cells. (C-D) Representative images and relative quantification of shctr, shAKTIP, and shTRF2 HeLa cells, stained for α-tubulin (green) and IST1 (red). For results in (D) at least 100 midbodies per condition were counted. Scale bar, 2.5μm. (TIF) [file pgen.1009757.s009.tif]
